# A high-precision synchronization and centroid-based framework for single-trial decoding of perceived natural speech

**DOI:** 10.3389/fncom.2026.1947069

**Published:** 2026-09-23

**Authors:** Cristian Y. Olivares, Norelli Schettini

**Affiliations:** 1 Department of Electrical and Electronics Engineering, Universidad del Norte, Barranquilla, Colombia; 2 Department of Mechatronics Engineering, Institución Universitaria de Barranquilla, Barranquilla, Colombia

**Keywords:** Brain-Computer Interface (BCI), FPGA synchronization, neural manifold, neuroprosthetics, phoneme decoding, single-trial EEG, Support Vector Machine (SVM)

## Abstract

Decoding natural speech from EEG is constrained by low signal-to-noise ratios, commonly addressed through event-related potential (ERP) grand averaging. However, this approach acts as a low-pass filter, obscuring trial-to-trial variability and high-frequency neural dynamics. To overcome these limitations, we propose an open-box, FPGA-synchronized framework for single-trial EEG analysis of Spanish vowels (/a/, /i/, /u/). The proposed architecture enforces sub-millisecond hardware determinism, eliminating software-induced temporal jitter and enabling robust extraction of high-frequency oscillatory features, including the Gamma band, without relying on averaged ERPs. This synchronization framework facilitates the separation of early exogenous sensory responses from late endogenous cognitive processing, while comprehensive ablation studies were performed to evaluate the effect of software-induced jitter on decoding performance. Results showed that introducing simulated jitter degraded classification performance to theoretical chance levels (33.20%), confirming hardware synchronization as a strict prerequisite for reliable single-trial decoding. The framework was further validated using a Centroid-PCA-SVM pipeline operating on a 16-channel spatial subset. Using the full proposed architecture, a grand mean classification accuracy of 67.70% was achieved, substantially exceeding the theoretical chance level of 33.33%. The across-participant mean of the maximum fold accuracies reached 83.00%, with individual validation folds achieving up to 90.00%. In contrast, zero-calibration leave-one-subject-out (LOSO) generalization remained at chance level (33.08%; κ_pooled_ = −0.0036), indicating limited cross-participant transferability and highlighting the current need for participant-specific calibration or alignment. Overall, these findings demonstrate that hardware-deterministic EEG acquisition enables the recovery of robust trial-by-trial brain dynamics that are typically obscured by conventional averaging approaches. By combining precise synchronization with low-latency algorithmic inference, the proposed framework establishes a foundation for real-time neural decoding and represents a stepping stone for practical brain-computer interfaces and advanced clinical monitoring applications.

## 1 Introduction

The decoding of speech processes from Electroencephalography (EEG) has predominantly relied on the extraction of Event-Related Potentials (ERPs) via grand averaging across multiple trials. While it has been hypothesized that single-trial data harbor sufficient discriminative information to characterize the underlying neural response to stimuli, leveraging such information remains a significant methodological challenge. Transitioning from ensemble averaging to single-trial analysis represents a fundamental departure from established analytical conventions, yet it is a critical requirement for the advancement of real-time Brain-Computer Interfaces (BCI). Historically, the widespread adoption of averaging has been obligated by the characteristically low Signal-to-Noise Ratio (SNR) of non-invasive neural recordings (Luck and Gaspelin, 2017).

Traditionally, researchers have worked with averaged ERPs to overcome these SNR limitations, often using closed-box systems that are not under the researcher's direct control. While averaging effectively cancels out stochastic background activity, it operates under the assumption of stationarity in both phase and latency—an assumption that is increasingly questioned in modern neuroscience, especially given the non-linear nature of neural oscillations (Sen et al., 2023). From a signal processing perspective, ensemble averaging acts as a low-pass filter that privileges phase-locked (evoked) activity while systematically canceling non-phase-locked (induced) oscillations. A critical methodological artifact arises when a neural component exhibits temporal jitter τ_*i*_ across trials. In such cases, the resulting average $\bar{x}\left(t\right)$ suffers from latency jitter-induced attenuation, or the smearing effect (Garaizar and Vadillo, 2014). For speech decoding, this blurring is particularly detrimental, as it masks fine-grained temporal dynamics and high-frequency components—specifically within the Gamma band (>30Hz)—which are essential for the encoding of phonological features (Silva et al., 2024; Tallon-Baudry and Bertrand, 1999).

Biophysically, the human brain does not generate identical responses to identical auditory stimuli. Trial-to-trial variability is not merely “noise” to be suppressed; it represents the dynamic state of neural networks, including attentional shifts and the refractory periods of the primary auditory cortex (Daniel et al., 2019; Ribeiro et al., 2024). By transitioning to Single-Trial Analysis, we move away from the static template-matching paradigm toward capturing the Neural Manifold—a high-dimensional representation where each trial constitutes a unique trajectory in the spatiotemporal feature space. This approach is vital for decoding Long Latency Responses (LLR), where endogenous processes (linguistic interpretation) introduce higher variance than early exogenous sensory captures. However, a fundamental obstacle in Single-Trial decoding is the temporal uncertainty introduced by software-based stimulus delivery, which typically ranges from 5 to 20 ms (Bridges et al., 2020). In an averaging scheme, this jitter is indistinguishable from neurogenic variability.

Brain-Computer Interfaces have traditionally relied on exogenous paradigms, such as Visual Evoked Potentials (VEPs) or P300 spellers, and endogenous tasks like Motor Imagery (MI). However, decoding speech from neural activity introduces a unique level of complexity. Unlike MI, which is localized in the motor cortex, or P300, which reflects a task-related oddball response, perceived speech involves a highly distributed network encompassing the primary auditory cortex, Wernicke's area, and the inferior frontal gyrus (Winkler et al., 2013; Wani, 2024). A critical distinction within this architecture is the separation of the neural response into two phases: exogenous components (early latencies such as N100 and P200 that reflect physical acoustic properties) (Fernández et al., 2022), and endogenous components or Long Latency Responses (occurring beyond 200 ms, modulated by linguistic expertise and phonological significance) (Barrett, 2009; Blumenfeld and Ranganath, 2019).

Recent studies have highlighted that the use of synthetic or “robotic” stimuli fails to activate the full linguistic manifold of the brain. To establish a rigorous phonological mapping and provide a canonical benchmarking paradigm, it is highly effective to utilize the fundamental vowel space. The cardinal vowels (/a/, /i/, /u/) constitute the vertices of the *vowel triangle*, effectively maximizing the Euclidean distance within the first and second formant (*F*1, *F*2) space, as illustrated in Figure 1.

**Figure 1:**
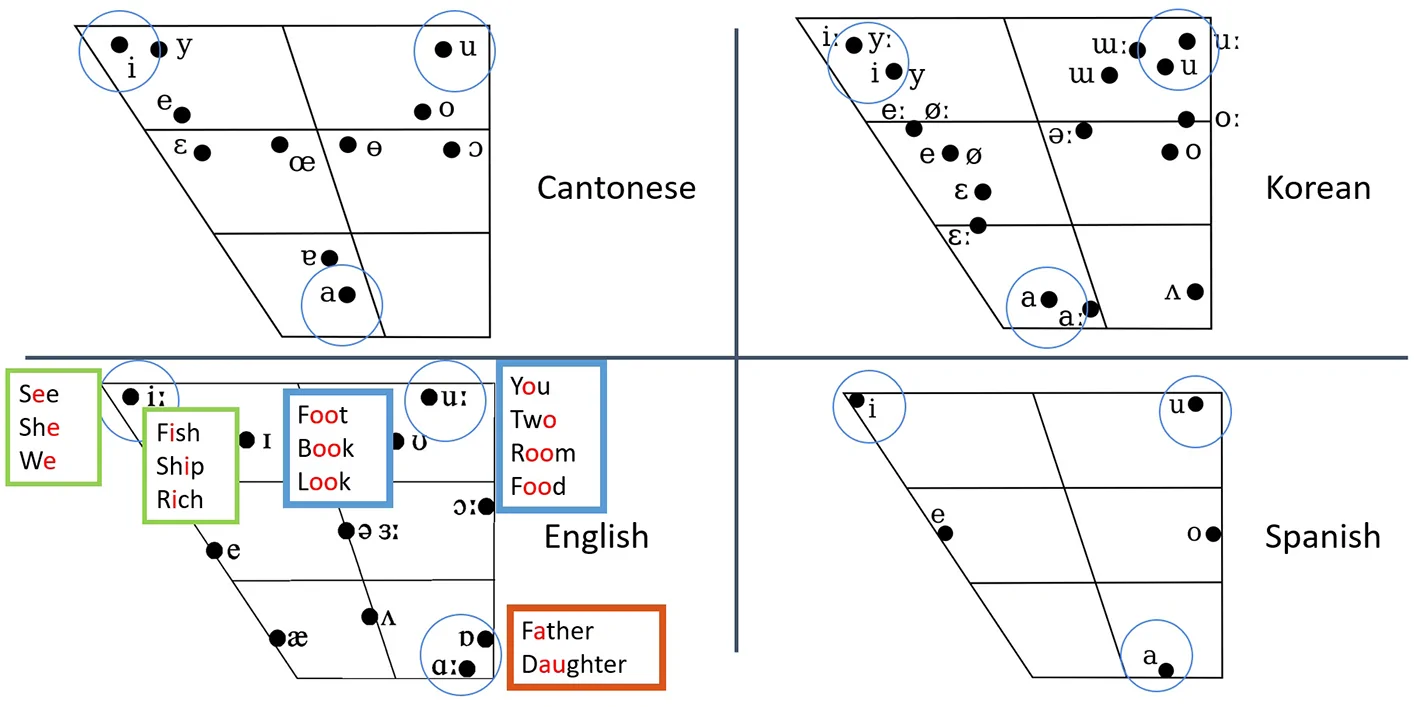
Vowel distribution within the phonetic space for Cantonese, Korean, English, and Spanish. Note that in most languages, at least three vowels occupy the extreme vertices (corners) of the map. Adapted from prior work (IPA Chart revised to 2015/2005; Kaya, 2018; International Phonetic Alphabet in IPA Kiel, 2026).

Using these phonological extremes ensures that the underlying Neural Manifold exhibits distinct spatiotemporal trajectories, thereby facilitating the evaluation of Single-Trial classifiers in a high-dimensional feature space. To ensure statistical power and replicability in the characterization of single-trial dynamics (Silva et al., 2024), our framework implements a rigorous protocol of 100 trials per class per subject using Perceived Uttered Natural Stimuli from native speakers (Rojas et al., 2016; Barajas et al., 2021; Panachakel and Ramakrishnan, 2021). This data density establishes the stability of Class Centroids and enables direct benchmarking against existing open-access datasets (Moinnereau et al., 2019).

To address these interconnected challenges, this study proposes a high-precision, FPGA-synchronized framework for the single-trial decoding of perceived natural speech. By enforcing sub-millisecond hardware determinism, this architecture effectively eliminates software-induced jitter, preserving the structural precision of high-frequency oscillatory features without the need for trial averaging. We validate this temporal transparency by introducing a novel Centroid-PCA-SVM pipeline applied to a robust high-density setup with 64 active electrodes (BioSemi ActiveTwo) recorded at 2048 Hz.

Unlike approaches based on regulated recurrent neural network reservoirs (Moinnereau et al., 2018) or frequency-band coupling models (Moinnereau et al., 2020), our framework simultaneously processes Perceived Uttered Natural Stimuli from native speakers while maintaining full temporal transparency. Instead of feeding raw data into deep neural networks—which may capitalize on temporal-autocorrelation features inherent to the stimuli and/or non-interpretable artifacts in jitter-prone datasets–our framework validates the neurophysiological integrity of each trial, isolating the exogenous sensory response from the endogenous linguistic processing.

Specifically, the contributions of this work are three-fold:

1. **A hardware-synchronized, open-box acquisition framework:** a hardware-deterministic acquisition architecture based on FPGA that achieves sub-millisecond synchronization, eliminating the confounding effects of temporal jitter on Single-Trial phase estimation.
2. **An interpretable single-trial feature extraction methodology:** a transparent feature engineering pipeline based on explicit feature (latency and phase) extraction post hard syncing, plus sub-band decomposition backed by a neuroscience and neurolinguistics criterion for electrode reduction and a distance-based method for class membership. This approach enables the identification of discriminative neural manifolds, all without the opacity typically associated with deep learning architectures.
3. **Experimental validation and spatiotemporal mapping:** we demonstrate that Perceived Uttered Natural Stimuli can be decoded with high precision (up to 90.00% for specific subjects, with a minimum of 40.00% and an average of 67.7%) using a high-resolution 2048 Hz dataset, while identifying a reduced subset of active channels that correspond to stable spatiotemporal windows of neural activity.

## 2 Materials and methods

### 2.1 Experimental design and participants

#### 2.1.1 Participants and ethics

For stimuli response evaluation, a total of 20 healthy volunteers (14 males, 6 females; age range 18–31), all native Spanish speakers with no history of neurological or hearing impairments, participated in the study. This participant sample size (*N* = 20) was determined based on prior *Single-Trial* EEG speech-decoding literature to ensure sufficient statistical power to classify neural responses to phonetic stimuli. Overall, the 20 subjects corresponded to a sample size of 100 single trials per vowel per subject, except for Subject 3, who completed 50 trials per vowel before voluntarily withdrawing during the inter-session rest period. All participants provided written informed consent prior to the experiment.

The study protocol was strictly conducted in accordance with the Declaration of Helsinki and was formally approved by the Institutional Ethics Review Board (Internal Act No. 273, July 28, 2022).

For stimuli recording, 5 healthy Spanish native speakers (3 males, 2 females) volunteered. There was no overlap between the voice recording volunteers and the subjects for stimulus-response assessment.

#### 2.1.2 Stimuli: natural vowel corpus and robustness

Speech decoding from EEG is typically done either by decoding synthetic formants or by decoding real speakers. Both approaches are based on the sounds located at the vocalic map corner as the starting point for stimulus selection due to the easy representation of these stimuli and how it is assumed that the brain processes this kind of stimuli, as those vowels are unequivocally different (distant) from each other, thus creating an adequate condition for discrimination.

Also, according to the vocalic map, the phonemes at the corners of the map show similar distributions across languages; this allows comparisons between speech decoders for languages with similar vowel maps, in this case, Cantonese, Korean, English, and Spanish. Thus, the main phonemes used as starting points to discriminate perceived speech are based on its first two frequency components (formants), regardless of the language of the study. For the Spanish case, the sounds /a/, /i/, or /u/ meet these requirements or their equivalents in Cantonese, Korean, or English, as is shown in Figure 1, allowing us to establish similarities, at least in the first stage of the hearing process.

The English language quadrant in Figure 1 includes examples of words with different meanings, but that sound similar to a non-native speaker—an exogenous response; due to the fact that, cognitively, the speech decoding of these sounds is entirely an endogenous process to native speakers, they are easily distinguishable.

Many studies suggest that participants' exposure to synthetic phonemes' pure components (formants) allows for discrimination in different languages when EEG signals are used (Kim et al., 2014; Fong et al., 2014) with a significant performance over 75% applying a one-vs-one classification over the three sound classes discussed above. Since the auditory cortex has a specific structure for processing the frequency of sounds perceived through the ear (Chittka and Brockmann, 2005), these detection rates are based on the activation or deactivation of these substructures; hence, a process highly related to a physiological response rather than a cognitive one: the exogenous process itself, discussed previously. In other words, multiple participants' brain activity will be the same when the stimuli are drawn from languages with similar vocalic maps, because the brain's response is exogenous, regardless of the participant's language.

In contrast, decoding EEG responses elicited by natural speech is generally less stable than decoding responses to isolated synthetic formants (Choi et al., 2015; Moinnereau et al., 2018; Liu and Ayaz, 2018). Across the broader single-trial literature, results remain highly heterogeneous, and reliable decoding is not consistently reproduced across participants, datasets, or validation protocols, even when reservoir networks, multilayer perceptrons, ensemble classifiers, and attention-based architectures are employed (Moinnereau et al., 2018; Hons et al., 2026; Wang et al., 2026; Li and Tao, 2026). This difficulty may arise because natural speech does not evoke an easily isolated activation or deactivation pattern within the auditory cortex. Nevertheless, perceived-stimulus information is present in distributed activity involving higher-order linguistic interpretation, reasoning, and memory access. These responses recruit multiple brain regions, emerge at longer latencies, and are therefore primarily associated with endogenous processing (Paulesu et al., 2000).

Recent studies nevertheless show a notable trend toward increasingly sophisticated methods for decoding perceived speech from EEG, combining spatiotemporal and frequency-domain features, time–frequency decomposition, neural networks, ensemble methods, and attention mechanisms (Moinnereau et al., 2018; Hons et al., 2026; Wang et al., 2026; Li and Tao, 2026). However, greater model complexity alone has not produced uniformly robust single-trial decoding, because outcomes remain sensitive to low signal-to-noise ratios, temporal alignment, inter-subject variability, class definitions, and validation design. Although Spanish-language datasets remain limited, studies conducted in languages with comparable vowel maps can provide descriptive reference points when the stimuli occupy similar positions in formant space and are presented to native speakers. Across these languages, auditory processing can be broadly characterized by an early, rapid response associated mainly with exogenous processing, followed by a later and more distributed response associated with endogenous linguistic and cognitive processing.

Accordingly, classifiers developed in languages other than Spanish can provide contextual reference values when they use comparable vowel classes and experimental conditions. Such comparisons should remain descriptive rather than be interpreted as direct benchmarks, because the reported outcomes depend on the dataset, cohort, class definitions, preprocessing, and validation protocol. Linguistic comparability is also strengthened when participants perceive stimuli in their native language, preserving the relationship between the acoustic token and its phonological interpretation (Paulesu et al., 2000). This consideration is particularly relevant because Spanish-language EEG datasets and validated single-trial speech classifiers remain scarce.

To evaluate our framework's robustness in ecological scenarios, we used a *Natural Vowel Corpus* rather than synthetic or MIDI-generated sounds, as is common in the literature. The stimuli consisted of the Spanish corner vowels (/a/, /i/, /u/), which are optimal for phonetic decoding due to their extreme positions in the F1-F2 formant space and their acoustic stationarity (Panachakel and Ramakrishnan, 2021).

The stimuli were recorded from 5 native speakers (3 males, 2 females)—who were entirely independent of the experimental cohort used for EEG data acquisition–to avoid speaker-specific and self-recognition bias. Vowels were extracted from natural continuous speech (e.g., the phrase “letter /a/”) using high-fidelity recording equipment (100 Hz–10 kHz frequency response, -38 dB sensitivity) and post-processed for normalization. Each trial consisted of a non-semantic cue sound, a 1-s rest period, and the target vowel sound, as presented in Figure 2.

**Figure 2:**
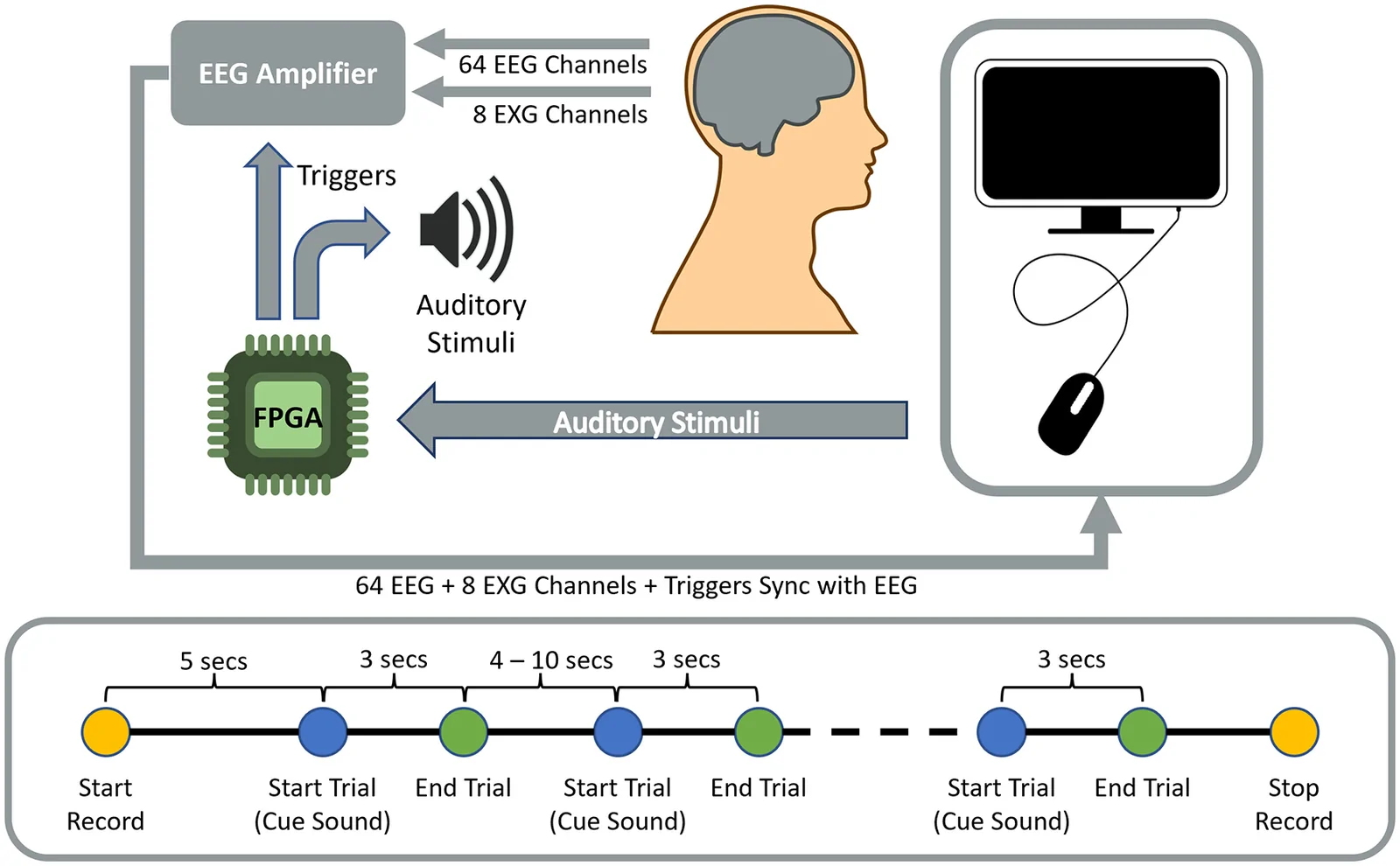
Experimental setup and temporal structure of the stimuli sequence.

To prevent anticipatory errors, habituation, and adaptation to a specific acoustic fingerprint, the experimental sequence was highly controlled. The inter-stimulus interval (ISI) and the trial order—including the random selection of which of the 5 native speakers' voices was presented for any given vowel trial—were randomized using the Mersenne Twister algorithm. Each subject completed a total of 340 trials (100 per vowel class, 20 white noise, and 20 silence), distributed across two recording sessions. The inclusion of this natural variability (intonation, duration, vocal timbre, and alternating speakers) ensures that the *Single-Trial* features captured by our FPGA-based system are representative of real-world generalized speech processing, rather than over-fitted to idealized, repetitive synthetic waveforms.

#### 2.1.3 FPGA-based hard synchronization

While software-triggered markers (e.g., Psychtoolbox, parallel-port pulses) are widely used in ERP experiments, they typically introduce 5 to 30 ms of non-deterministic latency jitter due to Operating System (OS) background scheduling and audio buffer delays. This variability critically undermines the temporal integrity required for single-trial analysis without grand averaging (Sanfins et al., 2018; Iwama et al., 2024). Specifically, such temporal jitter severely misaligns the phase of high-frequency EEG responses across trials; for instance, a 40 Hz Gamma oscillation has a period of 25 ms, meaning that a 10 ms timing error results in substantial phase cancellation.

To overcome this limitation and preserve high-frequency phase dynamics, we implemented a custom hardware-based synchronization unit on an Intel Cyclone IV EP4CE115 FPGA at the Register-Transfer Level (RTL), following the architecture detailed in Olivares and Schettini (2023). An analysis of the synthesized VHDL top-entity indicates that the internal logical latency is restricted to two 50 MHz clock cycles (40 ns) for threshold detection and state-machine triggering. Consequently, the maximum temporal jitter of the system is strictly bounded by the 48 kHz sampling period of the integrated audio codec, equating to a deterministic resolution of $T_{s}=\frac{1}{48000}\approx 20.83\;\mu \text{s}$.

The operational flow consists of three core stages:

1. **Acoustic onset detection:** an internal digital comparator and filtering stage identify the exact moment of stimulus onset by detecting when the audio signal crosses a predefined voltage threshold within the 40 ns logical window.
2. **Deterministic timestamping:** upon detection, the FPGA simultaneously routes the audio stream to the subject and generates a high-priority TTL pulse. This marker is embedded directly into the EEG bitstream, ensuring strict temporal alignment between the neural signal and the stimulus event.
3. **Experiment flow control:** the system utilizes physical push-buttons and an internal trial-counter register to automate the sequence of vowels (/a/, /i/, /u/), maintaining synchronization accuracy strictly within the < 21 μs hardware bound.

By enforcing this hardware-guaranteed determinism, the synchronization framework ensures that every single-trial epoch is temporally faithful to the acoustic stimulus. Eliminating OS-induced delays and bounding jitter to the microsecond scale preserves the trial-to-trial phase alignments of the recorded signals. This temporal precision is necessary for the subsequent extraction of phase-locked high-frequency oscillatory features, including the Gamma band, without relying on the low-pass filtering effects inherent to traditional grand averaging techniques.

#### 2.1.4 Data acquisition and recording setup

As presented in Figure 2, EEG data were acquired using a Biosemi Active Two system with 64 active electrodes arranged according to the international 10-20 system. Additionally, 8 external (EXG) channels were used: 7 for monitoring ocular (EOG) and cardiac (ECG) artifacts, and the 8th as a reference electrode placed on the earlobe. Signals were digitized at a high sampling rate of 2,048 Hz, providing the temporal resolution necessary to capture the phase-locked endogenous responses described previously.

To prevent anticipatory errors and habituation, the inter-stimulus interval (ISI) and trial order were randomized using the Mersenne Twister algorithm. Each subject completed a total of 340 trials (100 per vowel class, 20 white noise, and 20 silence), distributed across two recording sessions. To mitigate the effects of mental fatigue and ensure optimal cognitive engagement, a self-paced rest period was implemented between sessions. While participants were encouraged to resume only when fully ready to ensure comfort, the average rest duration was approximately 5 min. To monitor sustained attention and cognitive workload throughout the recording, a randomized behavioral validation task was integrated into the protocol. Specifically, an interactive dialog box appeared at unpredictable intervals, requiring the subject to identify the last stimulus heard. The stochastic nature of these queries introduced a “gamification” element, as participants were unaware of which trials would be evaluated, thereby enforcing continuous focus on the acoustic stimuli to ensure task accuracy.

The resulting dataset contains 340 single trials, with 100 per vowel per subject, plus the single trials corresponding to the response to the noise and silence samples. All trials were time-stamped via HardSyncing, and to the best of our knowledge, this represents the first single-trial dataset with hard synchronization stamps based on the subject's real sound perception, recorded from natural sounds uttered by native speakers, and with a large amount of single-trial samples per sound.

### 2.2 Signal preprocessing and biophysical artifact mitigation

Prior to hardware mapping and architectural synthesis, the continuous recorded raw EEG telemetry was preprocessed offline to maximize the physiological signal-to-noise ratio (SNR) and isolate true cortical speech-endogenous features. To prevent the injection of boundary discontinuities and filter-induced edge artifacts that inherently corrupt single-trial classification, all physiological and environmental artifact mitigation steps were executed systematically within the continuous time-series domain before any epoch segmentation.

Ocular, myogenic, and cardiac contamination were attenuated via an unsupervised, robust linear regression framework. To capture comprehensive noise dynamics, eight specialized external reference electrodes were employed: four dedicated to electrooculographic (EOG) and electromyographic (EMG) interference, three to cardiac activity (ECG), and one placed on the right earlobe acting as the system's structural reference for consistent baseline stability. Specifically, two EOG electrodes were positioned superior and inferior to the right orbit, and two EMG electrodes were placed immediately caudal to the left and right sphenoid processes to capture localized activity from the temporalis and masseter muscles.

Let *Y*_raw_(*c, t*) represent the continuous voltage amplitude of a cortical EEG channel *c* at time *t*, and let ${\text{X}}_{\text{art}}\left(t\right)={\left[x_{1}\left(t\right),\ldots ,x_{7}\left(t\right)\right]}^{T}$ denote the seven-dimensional vector of concurrent noise profiles recorded from the EOG, EMG, and ECG reference channels. The artifact-free neural signal *Y*_corrected_(*c, t*) was estimated by isolating and subtracting the linear projection of the noise space, as presented in Equation 1:


$$\begin{array}{l} Y_{\text{corrected}}\left(c,t\right)=Y_{\text{raw}}\left(c,t\right)-\beta_{c}^{T}{\text{X}}_{\text{art}}\left(t\right) \end{array}$$ (1)

where **β**_*c*_ represents the vector of channel-specific transfer coefficients calculated via ordinary least squares (OLS) minimization over the continuous unsegmented recording. Given the approximately linear coupling of these physiological potentials at the scalp level, this OLS approach provides an efficient noise-cancellation baseline. Because this regression matrix estimation is mathematically unsupervised and blind to phonemic class labels, it inherently preserves the absolute independence of the downstream validation boundaries. This structural attenuation effectively isolates true cortical potentials from ocular vectors, involuntary acoustic-myogenic tension, and cardiac transients that natively couple to natural speech perception.

Following continuous artifact subtraction, the corrected signal was bandpass filtered within an extended neuromodulatory spectrum (1.5–60 Hz). This upper cutoff boundary was selected to ensure the complete retention of high-frequency cortical oscillations while inherently attenuating standard electrical power-line interference (≥60 Hz). The filtering pipeline was parameterized using the fdesign object specifying a 4th-order digital Butterworth response, implemented via its butter method. To enforce a strict zero-phase distortion profile across all channels, the filter coefficients were applied bi-directionally (forward and backward) using the filtfilt function, preventing group delay artifacts and preserving the strict temporal alignments essential for high-precision single-trial decoding.

### 2.3 Sub-band decomposition and frequency analysis

Traditional ERP analysis often fails to capture the non-stationary dynamics of single-trial EEG due to the low Signal-to-Noise Ratio (SNR) of individual epochs. To address this, we adopt a multi-scale sub-band analysis framework, decomposing the signal into α (8–13 Hz), β (14–30 Hz), and γ (31–60 Hz) bands. This spectral characterization resolves previous definitional inconsistencies by aligning the upper boundary of the Gamma band with the global 60 Hz preprocessing filter. This allows for the identification of high-frequency features that remain robust even in the absence of consistent time-domain peaks in single trials (Biesmans et al., 2017).

The selection of these specific bands is grounded in their distinct functional roles during auditory perception:

- **Alpha (α):** associated with cortical inhibition and the modulation of attentional resources toward the auditory stimulus.
- **Beta (β):** linked to active linguistic processing and the maintenance of the cognitive set during phonological identification.
- **Gamma (γ):** critical for perceptual binding and high-level attention. Our results indicate that γ activity is paramount for distinguishing between semantic (vowels) and non-semantic (noise) content, particularly in fronto-temporal regions. The extraction of these dynamics is directly facilitated by the jitter-free environment provided by our FPGA synchronization.

By integrating this sub-band decomposition with spatial information, we observed differential neural activation patterns correlated with the temporal evolution of the response. As illustrated in Figure 3, brain activity significantly diverges between stimulus types at two critical latencies. The 100 ms mark corresponds to the N100 component, an exogenous response triggered by the abrupt onset of acoustic energy, temporally anchored by our FPGA-based trigger. Subsequently, the 175–200 ms window aligns with the P2 component, which exhibits high sensitivity to voice signals and endogenous semantic categorization (Winkler et al., 2013).

**Figure 3:**
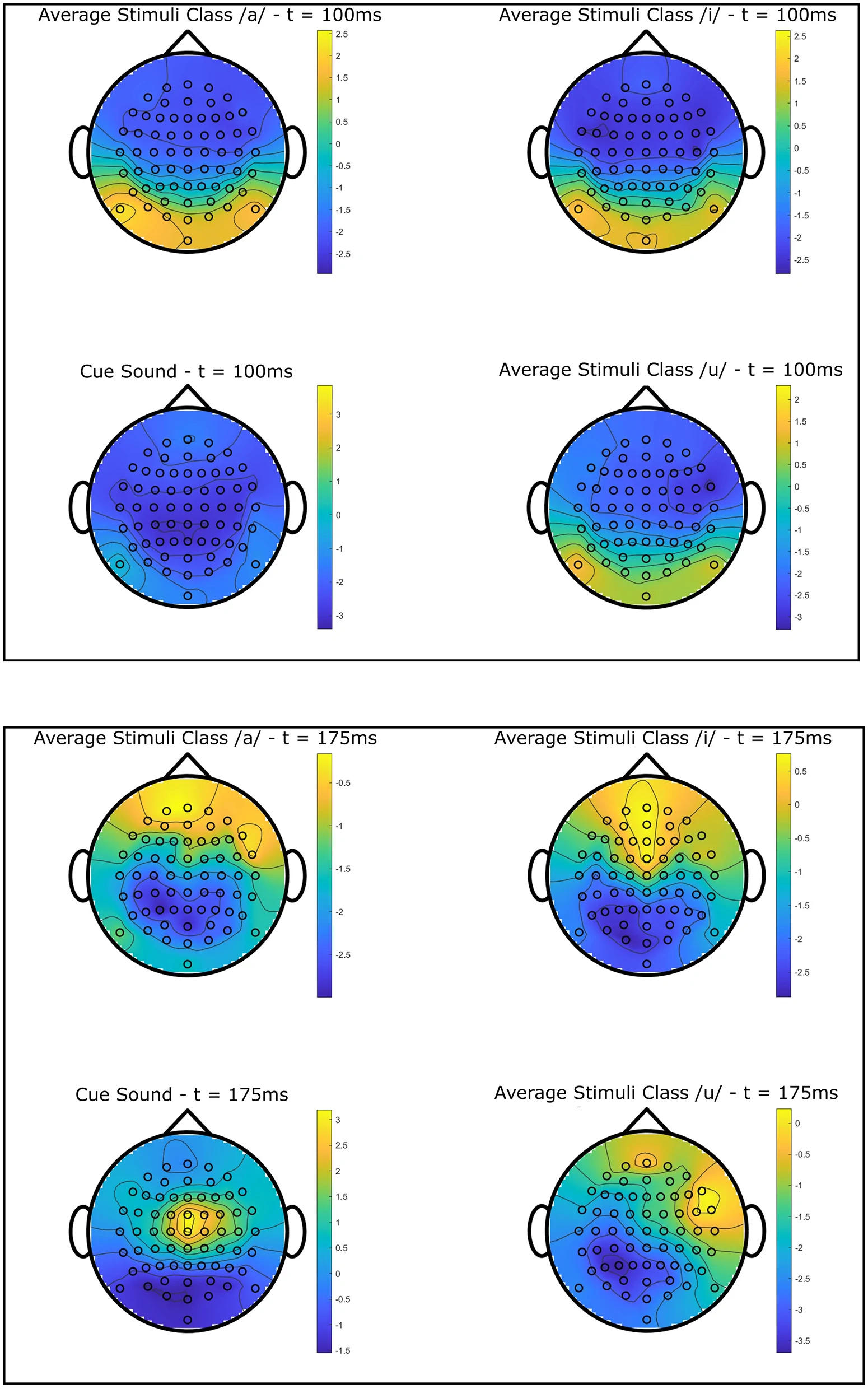
**(Top)** Brain activity to semantic and non-semantic stimuli around the 100ms timestamp. **(Bottom)** Brain activity to semantic and non-semantic stimuli around the 175ms timestamp.

This dual-domain approach (spectral-temporal) ensures that the feature vector provided to the classifier contains both the physical properties of the sound and the subsequent cognitive recognition of the vowel.

### 2.4 Centroid-based correlation algorithm

A significant barrier in single-trial EEG decoding is the presence of non-task-related physiological noise and environmental artifacts, which often mask the low-amplitude endogenous components associated with phonetic processing (Henry, 2014; Chittka and Brockmann, 2005). To mitigate this without resorting to traditional cross-subject ERP grand averaging—which inherently obliterates individual trial-by-trial variability and acts as an unintentional low-pass filter—we implemented an intra-subject Centroid-Based Correlation Algorithm.

It is crucial to structurally distinguish this algorithm from standard ERP grand averaging. Traditional grand averaging computes the mean of EEG epochs across multiple subjects and trials to yield a single, smoothed waveform for final analysis. This conventional approach effectively destroys single-trial phase dynamics and precludes real-time decoding. In contrast, our proposed algorithm strictly isolates the training and testing domains. The computed centroid (*C*_*k*_) serves exclusively as an intra-subject, class-specific matched filter derived solely from the training set, enabling the validation phase to remain strictly on un-averaged, single-trial data.

For each vowel class *k* ∈ {/*a*/, /*i*/, /*u*/}, the centroid *C*_*k*_ is computed within each specific frequency sub-band (α, β, γ). Mathematically, it is defined as the mean vector of all temporally synchronized trials within the training partition, and described in Equation 2:


$$\begin{array}{l} C_{k}=\frac{1}{n_{\text{train}}}\overset{n_{\text{train}}}{\underset{i=1}{\sum }}X_{i} \end{array}$$ (2)

where *X*_*i*_ is the voltage-time vector of an individual training trial and *n*_train_ denotes the total number of epochs for that class in the training subset.

Because the underlying framework enforces a high-precision, hardware-driven clock synchronization, the temporal alignment across trials is preserved at a microsecond level. Under this strict phase-locking condition, the linear summation in the time domain avoids the classic phase-cancellation artifacts observed in software-triggered ERPs. Instead, components with consistent phase relationships relative to the stimulus onset undergo constructive interference. Consequently, the resulting centroid vector *C*_*k*_ retains high-frequency oscillatory fidelity and implicitly encodes both the time-varying amplitude profiles and the dominant phase distribution of the specific phonemic class.

During feature extraction, an unseen, un-averaged single test trial is projected into a correlation space using these high-fidelity training centroids. Conversely, non-phase-locked background noise and stochastic oscillatory components undergo destructive interference during this projection, thereby enhancing the class-specific signal-to-noise ratio (SNR) while strictly preserving the unique transient dynamics necessary for true single-trial discrimination.

### 2.5 Spatiotemporal feature vector formulation

Building upon the robust class-specific centroids (*C*_*k*_) established through coherent phase-filtering, the subsequent step quantifies the spatio-temporal similarity between any unclassified single-trial observation and these derived templates across all frequency bands of interest.

Let $X_{i}^{\left(b\right)}$ represent the matrix of the *i*-th single-trial epoch filtered in the frequency band *b* ∈ {α, β, γ}. For this trial, a spatial Pearson correlation matrix ${\text{R}}_{i,k}^{\left(b\right)}$ is computed against each of the three class centroids $C_{k}^{\left(b\right)}$, as presented in Equation 3:


$$\begin{array}{l} {\text{R}}_{i,k}^{\left(b\right)}=\text{corr}\left(X_{i}^{\left(b\right)},C_{k}^{\left(b\right)}\right)\text{ for }k\in \left\{1,2,3\right\} \end{array}$$ (3)

This operation yields a set of correlation matrices that capture the degree of topographical and morphological alignment between the incoming trial and the expected evoked responses of each vowel class across the alpha, beta, and gamma rhythms.

To transform these multidimensional correlation structures into a format compatible with linear classification architectures, we apply a vectorization operator vec(·), which flattens each matrix ${\text{R}}_{i,k}^{\left(b\right)}$ into a one-dimensional column vector. Consequently, the comprehensive high-dimensional feature vector ${\text{f}}_{i}\in {\mathbb{R}}^{D}$ for the *i*-th trial is formed by the sequential concatenation of the vectorized correlation matrices for all classes *k* and frequency bands *b*, as described in Equation 4:


$$\begin{array}{l} {\text{f}}_{i}={\left[\text{vec}{\left({\text{R}}_{i,k}^{\left(b\right)}\right)}^{⊤}\right]}^{⊤}\;\forall \;b\in \left\{\alpha ,\beta ,\gamma \right\},\;k\in \left\{1,2,3\right\} \end{array}$$ (4)

Finally, to mitigate the curse of dimensionality and eliminate potential collinearity among the concatenated spatial features, Principal Component Analysis (PCA) is applied to the full training matrix composed of all **f**_*i*_ vectors. The eigenspace is truncated to retain only the subset of principal components that cumulatively explain at least 95% of the total variance, projecting both the training and testing datasets into an optimal lower-dimensional manifold prior to classification.

### 2.6 Electrode selection and dimensionality reduction

To optimize the signal-to-noise ratio (SNR) and minimize the computational overhead for real-time applications, the overall dimensionality of the system was managed through a dual-stage strategy: an initial physiological spatial filtration, followed by the previously defined statistical compression (PCA).

Traditional 64-channel setups capture significant volume conduction and redundant information that typically leads to overfitting in single-trial classification. Therefore, the first stage involved a spatial reduction based on the observed *Event-Related Desynchronization* (ERD) in the α band and *Event-Related Synchronization* (ERS) in the β and γ bands. By integrating the spatial power distribution across trials, we identified a consistent pattern of electrode behavior associated with semantic vowel processing (see Figure 4). Electrodes were classified as “Top Performers” if they consistently exhibited minimum power in the α band (indicating attentional engagement) and peak power in the β and γ bands (reflecting endogenous phonological decoding).

**Figure 4:**
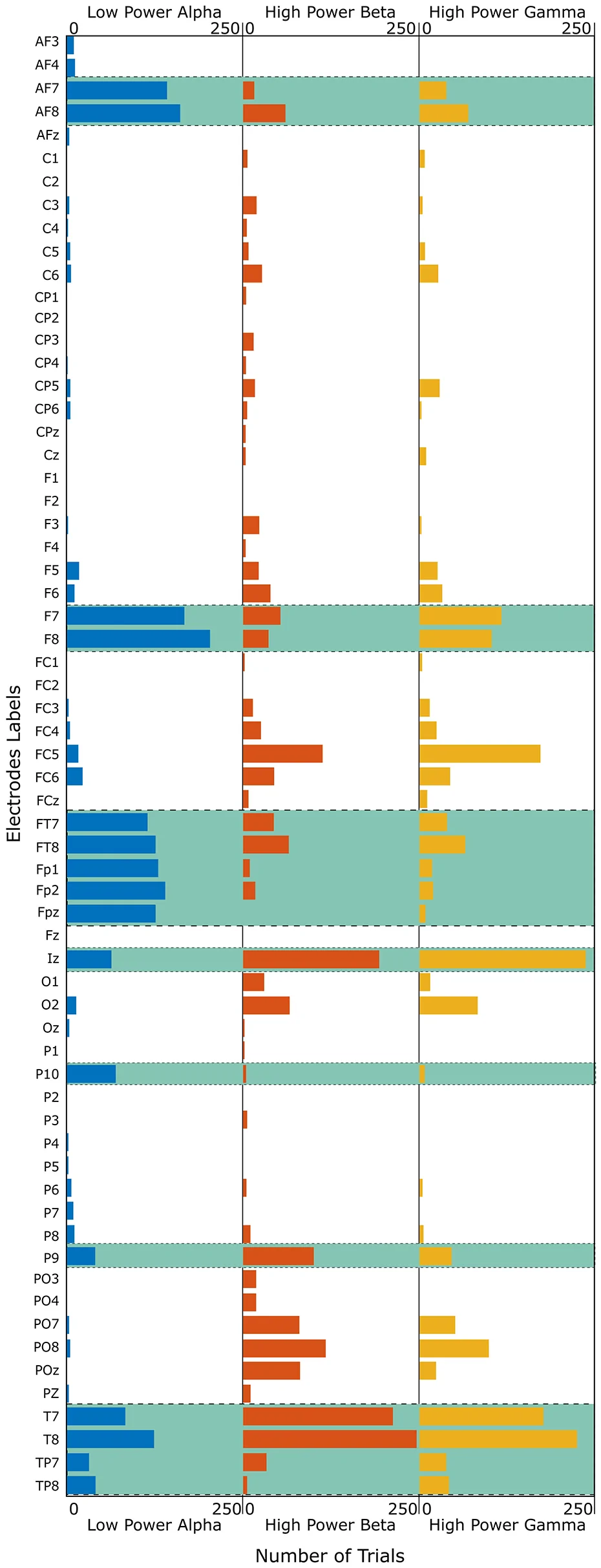
Spatial distribution of spectral power across trials for semantic stimuli. The histogram represents the cumulative count of electrodes exhibiting a simultaneous *Event-Related Desynchronization* (ERD) in the α band and *Event-Related Synchronization* (ERS) in the β and γ bands. High-performing electrodes (highlighted in green) identify the cortical regions with the highest consistency in semantic processing across subjects.

This neurophysiologically driven procedure reduced the input space from 64 to 16 optimized channels (a 75% reduction in spatial dimensionality). The selected electrodes—**AF8, F8, AF7, F7, Fp2, Fp1, FpZ, FT7, FT8, T8, P10, T7, TP8, Iz, P9, and TP7**—are strategically localized in regions critical for auditory processing and language comprehension, as shown in Figure 5. Notably, this subset captures activity from the auditory cortex and the lateral prefrontal cortex, areas previously linked to superior cognitive activities and semantic categorization (Morillon et al., 2022; Moerel et al., 2012).

**Figure 5:**
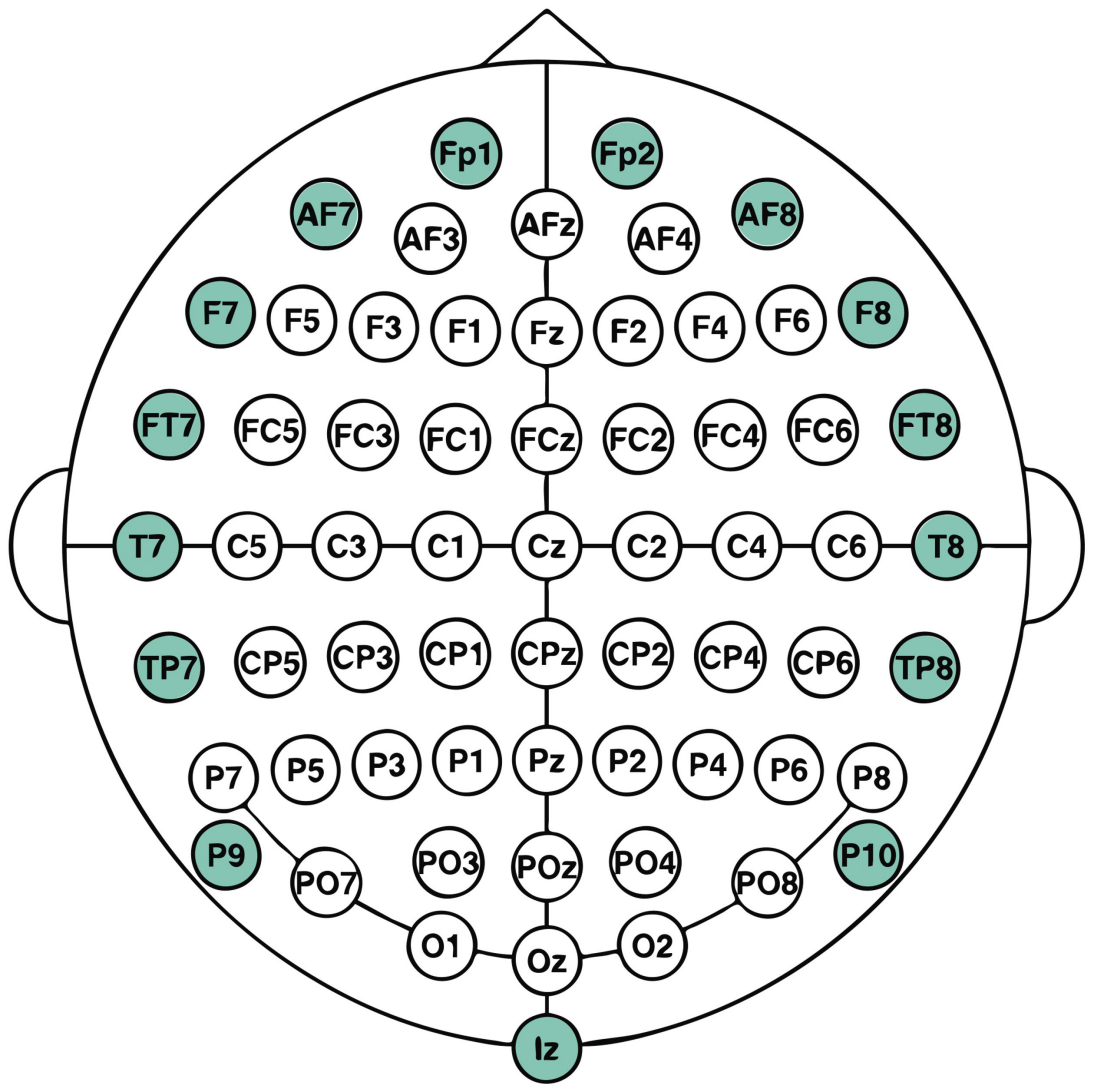
Topographic map (10–20 system) of the 16 optimized electrodes selected for dimensionality reduction. The subset focuses on fronto-temporal and occipital regions (e.g., FT7, FT8, T7, T8, and Iz), aligning with the neural circuitry involved in auditory phonological mapping and superior cognitive vowel categorization.

The integration of this spatial optimization with the centroid-based feature formulation establishes the structural prerequisite for the final PCA stage. As introduced in Section 2.5, PCA is applied to the vectorized correlation matrices (**f**_*i*_) rather than the raw EEG epochs. This specific architectural sequence is mathematically critical to the pipeline's success: applying PCA directly to raw, high-density single-trial EEG primarily generates principal components that maximize background physiological stochasticity (noise). In contrast, by restricting the spatial domain to the 16 optimized channels and subsequently applying PCA strictly to the centroid-projected feature vectors, the algorithm effectively compresses the preserved phase-locked phonological variance. This sequential combination guarantees the elimination of redundant collinearities without modeling random noise.

Refining the feature extraction through this optimized spatial subset highlights subtle latency variations and phase shifts that are typically masked in traditional ERP averaging. As illustrated in the Phase Spectrum analysis (Figure 6), the use of the Discrete Fourier Transform (DFT) over the window of interest reveals class-specific local maxima. These phase-based features, strictly preserved by the FPGA synchronization, provide high discriminative power for *Single-Trial* decoding, capturing the *endogenous* neural dynamics that differentiate between the phonemes /a/, /i/, and /u/.

**Figure 6:**
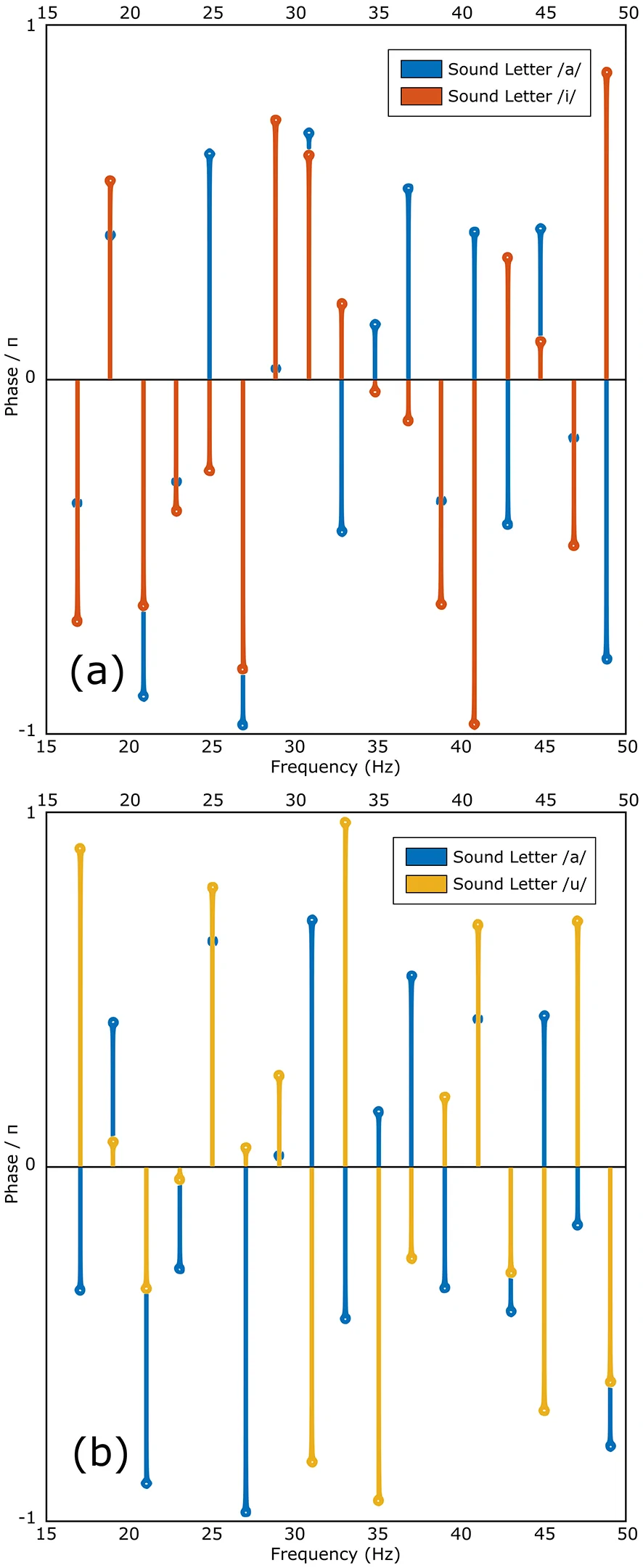
Average Phase Spectrum comparison for semantic contrasts (/a/ vs. /i/ and /a/ vs. /u/) within the combined β+γ bands (>15 Hz). **(a)** Phase vector for /a/ vs. /i/. **(b)** Phase vector for /a/ vs. /u/. Note the distinct local maxima and phase shifts preserved by the FPGA-based hardware synchronization, which provide discriminative *Single-Trial* features that are typically lost in traditional time-domain averaging.

## 3 Results

To validate the proposed framework, it is critical to assess the discriminative power of the class features obtained using our methodology. These feature vectors could represent a paradigm shift in the use of single-trial EEG for common BCI applications by successfully bypassing the traditional reliance on grand-averaged responses. Figure 7 illustrates the overall system schema, detailing the training and testing pipelines of the classifier within our proposed framework.

**Figure 7:**
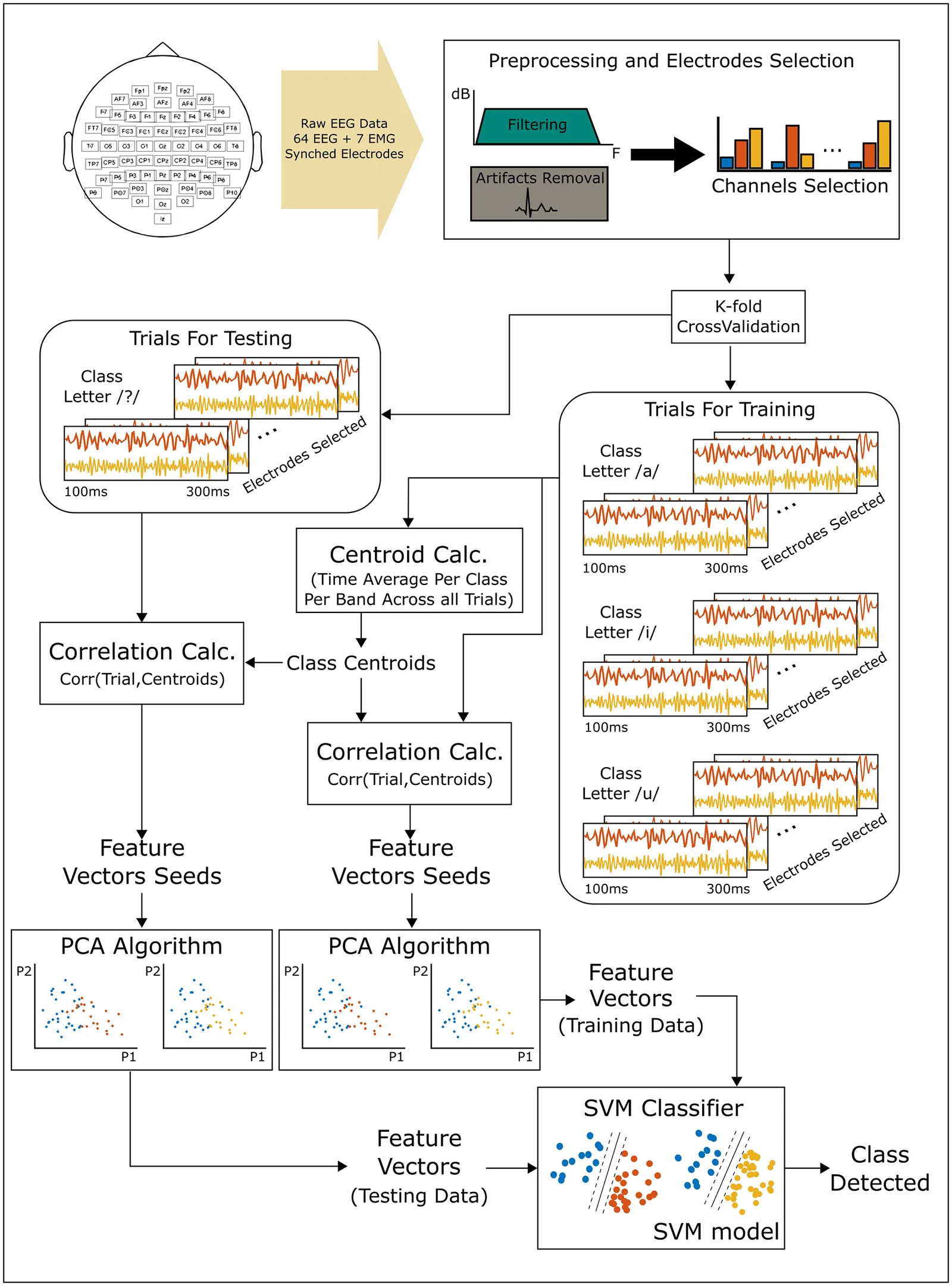
General System Schema implemented to train the classifier and to classify each new test trial sample.

To rigorously evaluate the framework's efficacy, we benchmarked the extracted features against standard decoding pipelines. Specifically, we implemented a single-trial Spanish vowel speech decoder, designed to test whether our sub-band centroid-based feature space captures sufficient neural information to perform robust classification in the complete absence of ensemble averaging.

To independently assess the contribution of each module of our pipeline, ablation studies were performed and the results are presented in Section 3.3

### 3.1 Spatiotemporal feature mapping

The neurophysiological validity of the framework is grounded in the mapping of spatiotemporal characteristics across different frequency bands. Principal Component Analysis (PCA) was employed to visualize how the framework organizes noisy trials into discriminable clusters.

#### 3.1.1 Sub-band dynamics and electrode optimization

The response to semantic stimuli was characterized by a distinct spectral signature across three sub-bands:

- **Alpha (α, 8–13 Hz):** consistent Event-Related Desynchronization (ERD) was observed, indicating the allocation of attentional resources toward the auditory stimulus.
- **Beta (β, 14–30 Hz) and Gamma (γ**, **≥** **31 Hz):** these bands exhibited Event-Related Synchronization (ERS) linked to endogenous linguistic processing and phonological identification.

By applying a criterion of minimum Alpha power and maximum Beta/Gamma power, the input dimensionality was reduced by 75% (from 64 to 16 optimized electrodes). The selected subset (including FT7, FT8, T7, T8, and Iz) aligns with the primary auditory cortex and lateral prefrontal regions, areas traditionally involved in semantic categorization and superior cognitive activities. This targeted electrode selection further explains why the baseline approach—which did not employ such frequency-band optimization—failed to extract discriminable information from the synchronized signals.

#### 3.1.2 Neural manifold and cluster organization

To address the challenges of low SNR in single trials, the system utilized Feature Vector Seeds derived from the Pearson correlation (*r*) between individual trials and class centroids (*C*_*k*_).

The PCA projection (Figure 8) confirms that while raw signals exhibit total overlap—consistent with the poor performance observed in the baseline evaluation—the correlated feature vectors reveal well-defined clusters for each phoneme. This transformation effectively captures the trajectory of the “Neural Manifold” within the 100–300 ms window. The first three principal components were used for graphical presentation, proving that the discriminative information for vowels is not stochastic but resides in stable spatiotemporal windows corresponding to Long Latency Responses (LLR). The contrast between the overlapping raw data clusters and the well-separated final feature vectors provides visual corroboration of the quantitative improvement observed when comparing the baseline (35.80%) and proposed (67.70%) mean accuracies.

**Figure 8:**
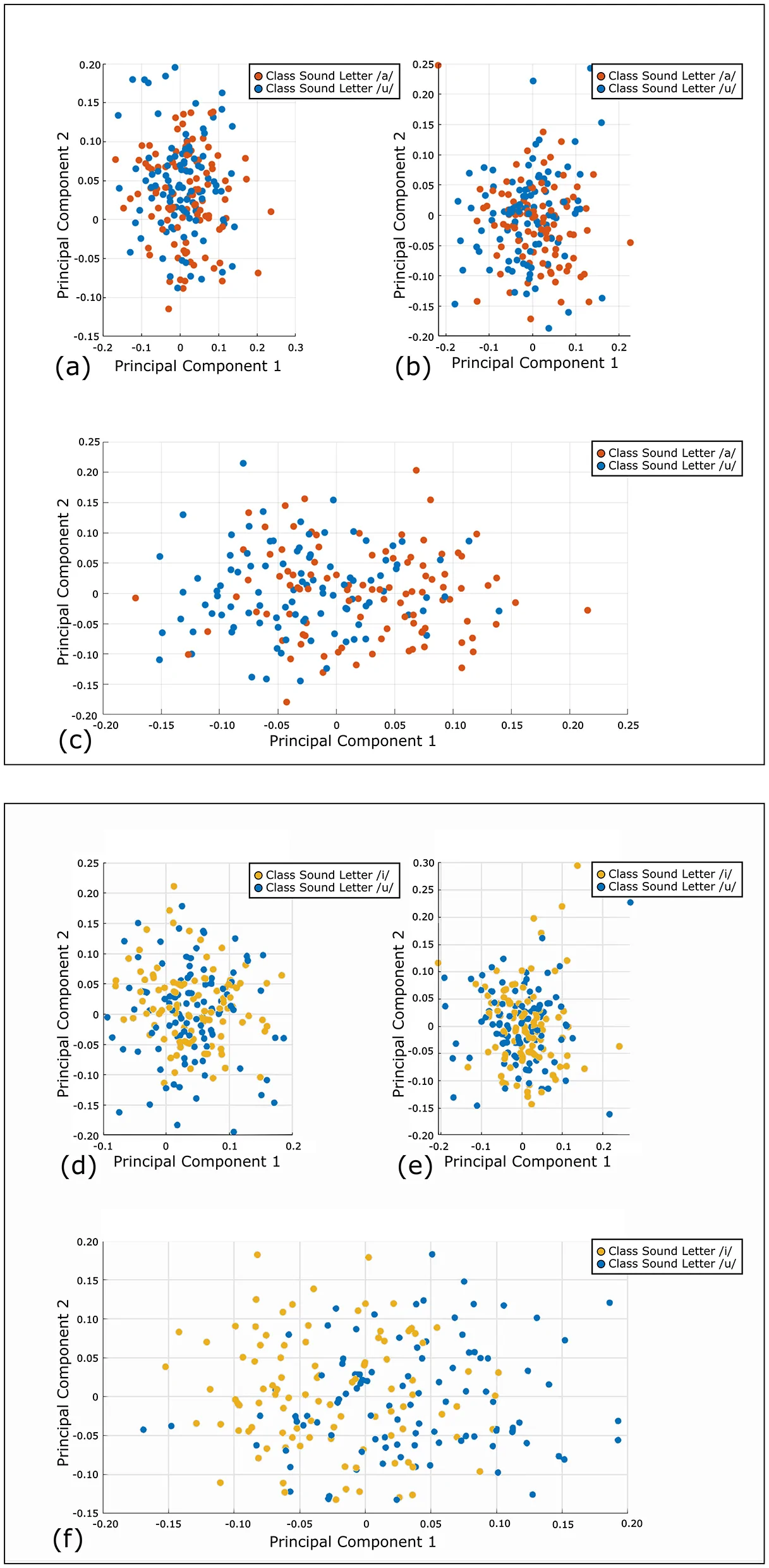
Principal Component Analysis (PCA) of the feature space transition for Single-Trial vowel decoding. The clusters illustrate class separability at different processing stages. **(Top)** Binary classification of /a/ vs. /i/: **(a)** raw windowed data after k-folding, **(b)** sub-band filtered signals (Beta + Gamma, >15 Hz), and **(c)** proposed Feature Vector Seeds derived from centroid correlation. **(Bottom)** Binary classification of /i/ vs. /u/: **(d)** raw windowed data, **(e)** sub-band filtered signals, and **(f)** final feature vectors. Note the progressive cluster consolidation in **(c)** and **(f)**, demonstrating the efficacy of the correlation-based dimensionality reduction.

### 3.2 Feature space isolation and leakage-free cross-validation

To evaluate the generalization performance of the proposed Centroid-Based Correlation Algorithm and the downstream SVM classifier, a rigorous *K*-fold cross-validation (*K* = 5) scheme was implemented on a per-subject basis to characterize intra-individual decoding stability. In every fold, 80% of the participant's EEG trials were allocated to training and the remaining 20% were reserved for testing. To maintain strict separation between these partitions and minimize the risk of data leakage, all dimensionality-reduction parameters and class centroids were computed exclusively from the training folds for each participant.

Let **X**_train_ represent the matrix containing the single-trial segments from the *K* − 1 folds allocated for training, and let **X**_test_ denote the completely unseen trials from the remaining single fold reserved for testing. The spatial optimization via Principal Component Analysis (PCA) was parameterized exclusively on the training matrix using Singular Value Decomposition (SVD), which is described in Equation 5:


$$\begin{array}{l} {\text{X}}_{\text{train}}-\mu_{\text{train}}=\text{U}\Sigma {\text{V}}^{T} \end{array}$$ (5)

where **μ**_train_ is the empirical row-mean vector calculated solely from **X**_train_, and **V** represents the orthogonal matrix of principal component coefficients (coeff).

The optimal number of latent dimensions *d* (num_comp) was dynamically selected in each cross-validation iteration to satisfy a strict cumulative variance threshold, calculated as presented in Equation 6:


$$\begin{array}{l} d=\arg\underset{m}{\min}\left(\overset{m}{\underset{i=1}{\sum }}\lambda_{i}\geq 95\%\right) \end{array}$$ (6)

where λ_*i*_ represents the percentage of variance explained by the *i*-th eigenvalue. The low-dimensional training feature space was then mapped as presented in Equation 7:


$$\begin{array}{l} {\text{X}}_{\text{train, pca}}=\left({\text{X}}_{\text{train}}-\mu_{\text{train}}\right){\text{V}}_{1:d} \end{array}$$ (7)

The class centroids defining the core reference patterns of the Centroid-Based Correlation Algorithm were computed exclusively over the coordinates of **X**_train, pca_ for each vowel class.

To guarantee that no predictive priors or empirical distributions from the testing set contaminated the training phase, the test partition **X**_test_ was treated as completely blind out-of-sample data, as shown in Equations 8 and 9, where the projection of the test trials into the pre-established latent space was performed strictly using the training-derived transformation matrices:


$$\begin{array}{l} {\text{X}}_{\text{test, centered}}={\text{X}}_{\text{test}}-\mu_{\text{train}} \end{array}$$ (8)

$$\begin{array}{l} {\text{X}}_{\text{test, pca}}={\text{X}}_{\text{test, centered}}{\text{V}}_{1:d} \end{array}$$ (9)

By projecting **X**_test_ through **μ**_train_ and **V**_1:*d*_ rather than re-estimating the coefficients or the mean globally, the validation boundaries remain independent of the held-out EEG epochs. This conservative protocol substantially reduces the possibility that the performance gap between the baseline (35.80%) and proposed (67.70%) methods arises from feature-transformation or centroid leakage.

Regarding acoustic-stimulus allocation, the five native speakers who recorded the auditory tokens were entirely distinct from the 20 participants whose EEG responses were analyzed, thereby avoiding speaker–participant identity overlap. Because the five stimulus voices were randomly interspersed throughout the recording sessions, tokens produced by the same speaker could appear in both the training and testing folds. Nevertheless, these partitions contained mutually exclusive EEG epochs, and the classifier received neurophysiological responses rather than audio waveforms as input.

Accordingly, this design minimizes epoch-level leakage and evaluates generalization to unseen EEG trials from a calibrated participant while retaining the same pool of stimulus speakers. It should not be interpreted as speaker-independent or token-independent generalization to previously unheard voices or recordings. A future validation in which stimulus speakers or individual audio tokens are held out would provide a stricter test of whether the decoded representation is invariant to speaker identity and token-specific acoustics.

### 3.3 Ablation Study: quantifying the isolated contributions of each module

To rigorously isolate and quantify the specific performance gains provided by each technical component of our architecture, we consolidated our evaluations into a comprehensive ablation study. The evaluation follows a systematic, step-by-step progression across five distinct configurations to prove that each module plays an irreplaceable role in single-trial decoding.

#### 3.3.1 Performance metrics and pooled Cohen's kappa

To ensure a robust and unbiased performance assessment across all ablation tiers, both single-trial classification accuracy and chance-corrected agreement (Cohen's κ) were computed from out-of-fold predictions. Accuracy was first calculated for each participant and configuration and was then summarized across subjects as the mean ± standard deviation (SD). For the cohort-level agreement statistic, the subject-level confusion matrices were summed within each configuration to obtain a single pooled confusion matrix **N** = [*n*_*ij*_], where rows denote the true vowel labels and columns denote the predicted labels. Thus, κ_pooled_ was calculated from all pooled test decisions rather than as the arithmetic mean of the individual-subject κ values.

Let $N=\overset{K}{\underset{i=1}{\sum }}\overset{K}{\underset{j=1}{\sum }}n_{ij}$ be the total number of pooled test decisions for the *K* = 3 target classes (/a/, /i/, /u/), and define the cell and marginal proportions as described in 10.


$$\begin{array}{l} p_{ij}=\frac{n_{ij}}{N},\;p_{i+}=\overset{K}{\underset{j=1}{\sum }}p_{ij},\;p_{+j}=\overset{K}{\underset{i=1}{\sum }}p_{ij}. \end{array}$$ (10)

The observed and chance-expected agreements are calculated using Equation 11.


$$\begin{array}{l} P_{o}=\overset{K}{\underset{i=1}{\sum }}p_{ii},\;P_{e}=\overset{K}{\underset{i=1}{\sum }}p_{i+}p_{+i}, \end{array}$$ (11)

and the pooled coefficient is therefore calculated as described in Equation 12


$$\begin{array}{l} \kappa_{\text{pooled}}=\frac{P_{o}-P_{e}}{1-P_{e}}, \end{array}$$ (12)

following the definition of Cohen's kappa (Cohen, 1960).

The standard error was obtained from the non-null large-sample variance estimator of Fleiss, Cohen, and Everitt (Fleiss et al., 1969). For the pooled proportions defined above, its components are described in Equations 13–15.


$$\begin{array}{l} V_{1}=\overset{K}{\underset{i=1}{\sum }}p_{ii}{\left[\left(1-P_{e}\right)-\left(p_{i+}+p_{+i}\right)\left(1-P_{o}\right)\right]}^{2}, \end{array}$$ (13)

$$\begin{array}{l} V_{2}={\left(1-P_{o}\right)}^{2}\underset{i\neq j}{\sum }p_{ij}{\left(p_{+i}+p_{j+}\right)}^{2}, \end{array}$$ (14)

$$\begin{array}{l} V_{3}={\left(P_{o}P_{e}-2P_{e}+P_{o}\right)}^{2}, \end{array}$$ (15)

yielding Equation 16.


$$\begin{array}{l} SE\left(\kappa_{\text{pooled}}\right)=\sqrt{\frac{V_{1}+V_{2}-V_{3}}{N{\left(1-P_{e}\right)}^{4}}}. \end{array}$$ (16)

When required, the two-sided 95% confidence interval was calculated as κ_pooled_ ± 1.96*SE* and truncated to the admissible interval [−1, 1]. This dual-metric evaluation—across-subject mean accuracy with SD alongside κ_pooled_ with SE—accounts for the chance agreement implied by the empirical class marginals and provides a consistent basis for comparing the architectural tiers below.

#### 3.3.2 Group 1: the critical necessity of hardware synchronization (software jitter baseline)

To address the fundamental premise of our framework–that sub-millisecond hardware determinism is strictly required for single-trial high-frequency decoding–we first established a baseline evaluating the impact of traditional software-based synchronization. To mimic the latency variability inherent to standard USB-based triggers and non-real-time operating systems, a synthetic temporal jitter (±30 ms) was artificially introduced into the data prior to the feature extraction pipeline.

As detailed in Table 1, the introduction of this temporal jitter completely degrades the system's decoding capability, even when utilizing the full Centroid-PCA architecture. The global mean accuracy collapsed to 33.20% (κ = −0.001), which is statistically indistinguishable from the theoretical chance level (33.33% for a 3-class paradigm). This severe phase misalignment obliterates the phase-locked high-frequency spatial patterns, rendering the algorithm unable to extract discriminative features. These baseline results decisively prove that algorithmic refinement alone is insufficient if the structural precision of the signal is compromised; sub-millisecond hardware synchronization is a mandatory prerequisite.

**Table 1:** Ablation group 1: baseline decoding performance under simulated software-induced temporal jitter (±30 ms).

Subject	Accuracy	Kappa (κ)
1	0.271	−0.094
2	0.348	0.023
3	0.300	−0.050
4	0.398	0.098
5	0.355	0.033
6	0.353	0.029
7	0.344	0.016
8	0.353	0.030
9	0.362	0.043
10	0.288	−0.069
11	0.318	−0.024
12	0.312	−0.033
13	0.327	−0.010
14	0.373	0.059
15	0.350	0.025
16	0.312	−0.033
17	0.345	0.018
18	0.342	0.013
19	0.322	−0.018
20	0.265	−0.103
**Summary**	**0.332** **±0.034**	**−0.001 (0.007)**

Accuracy is reported as a proportion and individual values are rounded to three decimal places. In the final row, accuracy is summarized as the across-subject mean ± standard deviation (SD), whereas Cohen's κ is reported as the pooled estimate with its standard error in parentheses, κ_pooled_ (SE). This ablation applies simulated software-induced temporal jitter (±30 ms) to the full classification pipeline. The resulting mean accuracy (0.332) and pooled κ (−0.001) indicate performance at the theoretical chance level (0.333) and no agreement beyond chance.

#### 3.3.3 Group 2: hardware synchronization alone is insufficient (broadband raw EEG)

Having established the critical necessity of hardware-level synchronization, we next evaluated whether this precise temporal alignment alone is sufficient for single-trial decoding. To test this, we processed the fully FPGA-synchronized broadband EEG dataset using a standard classification pipeline, bypassing our specific sub-band and Centroid-Based Correlation algorithms entirely.

As shown in Table 2, while hardware determinism guarantees phase integrity, applying traditional classification approaches to raw synchronized data still fails to decode speech dynamics on a single-trial basis. The global mean accuracy only reached 35.80% (κ = 0.035 for 16 channels and 35.7% for 64 channels), remaining near chance levels. This reveals that the low signal-to-noise ratio inherent to un-averaged single trials masks discriminable neural representations, motivating the need for advanced feature-space refinement.

**Table 2:** Ablation group 2: decoding performance on FPGA-synchronized broadband EEG without subband decomposition, centroid, or PCA processing.

Subject	16CH RAW ALL		64CH RAW ALL	
	Accuracy	Kappa (κ)	Accuracy	Kappa (κ)
1	0.357	0.035	0.325	−0.013
2	0.345	0.018	0.334	0.001
3	0.460	0.190	0.455	0.183
4	0.365	0.048	0.381	0.071
5	0.367	0.050	0.355	0.033
6	0.340	0.010	0.355	0.033
7	0.386	0.079	0.404	0.106
8	0.349	0.024	0.403	0.105
9	0.364	0.046	0.369	0.054
10	0.403	0.105	0.390	0.085
11	0.348	0.021	0.333	0.000
12	0.349	0.024	0.367	0.050
13	0.311	−0.034	0.342	0.013
14	0.350	0.025	0.334	0.001
15	0.379	0.069	0.325	−0.013
16	0.317	−0.025	0.353	0.030
17	0.321	−0.019	0.299	−0.051
18	0.343	0.015	0.305	−0.043
19	0.370	0.055	0.367	0.050
20	0.358	0.036	0.334	0.001
**Summary**	**0.359** **±0.033**	**0.035 (0.007)**	**0.357** **±0.037**	**0.031 (0.007)**

Accuracy is reported as a proportion and individual values are rounded to three decimal places. In the final row, accuracy is summarized as the across-subject mean ± standard deviation (SD), whereas Cohen's κ is reported as the corresponding pooled estimate with its standard error in parentheses, κ_pooled_ (SE). This ablation compares 16- and 64-channel FPGA-synchronized broadband EEG without subband decomposition, centroid construction, or PCA. The mean accuracies (0.359 and 0.357) and small pooled κ values (0.035 and 0.031) indicate performance only marginally above the 0.333 chance level.

#### 3.3.4 Group 3: spectral isolation without spatial-temporal mapping (sub-bands only)

Progressing to the third tier, we evaluated whether isolating frequency bands (Alpha, Beta, and Gamma) on FPGA-synchronized data without computing centroids or PCA could achieve single-trial separability.

As shown in Table 3, Group 3 decoding performance remained close to chance across all individually analyzed frequency bands. Alpha-band configurations achieved 36.6%–36.8% accuracy (κ_pooled_ = 0.045–0.050), Beta-band configurations achieved 34.4%–35.0% (κ_pooled_ = 0.014–0.025), and Gamma-band configurations fell slightly below chance at 32.5%–32.9% (κ_pooled_ ≤ −0.008). Overall, isolating individual frequency bands was insufficient without an effective spatiotemporal feature-mapping stage.

**Table 3:** Ablation group 3: decoding performance on FPGA-synchronized EEG using separate frequency bands without centroid or PCA processing.

Subject	16CH RAW ALPHA		64CH RAW ALPHA		16CH RAW BETA		64CH RAW BETA		16CH RAW GAMMA		64CH RAW GAMMA	
	Accuracy	Kappa (κ)	Accuracy	Kappa (κ)	Accuracy	Kappa (κ)	Accuracy	Kappa (κ)	Accuracy	Kappa (κ)	Accuracy	Kappa (κ)
1	0.328	−0.009	0.317	−0.025	0.353	0.029	0.355	0.033	0.357	0.035	0.328	−0.009
2	0.361	0.041	0.306	−0.041	0.370	0.055	0.358	0.038	0.297	−0.055	0.285	−0.073
3	0.432	0.148	0.463	0.195	0.360	0.040	0.393	0.090	0.365	0.048	0.398	0.098
4	0.389	0.084	0.365	0.048	0.374	0.061	0.388	0.081	0.347	0.020	0.320	−0.020
5	0.378	0.066	0.347	0.020	0.360	0.040	0.360	0.040	0.342	0.013	0.330	−0.005
6	0.311	−0.034	0.344	0.016	0.377	0.065	0.313	−0.030	0.322	−0.017	0.334	0.001
7	0.403	0.105	0.372	0.058	0.378	0.068	0.383	0.075	0.359	0.039	0.342	0.013
8	0.353	0.030	0.362	0.043	0.344	0.016	0.390	0.085	0.325	−0.013	0.303	−0.046
9	0.360	0.040	0.385	0.078	0.346	0.019	0.303	−0.046	0.310	−0.035	0.319	−0.021
10	0.511	0.266	0.489	0.234	0.328	−0.009	0.301	−0.049	0.323	−0.016	0.317	−0.025
11	0.345	0.018	0.349	0.024	0.343	0.014	0.343	0.014	0.333	0.000	0.318	−0.022
12	0.336	0.004	0.358	0.038	0.319	−0.021	0.360	0.040	0.322	−0.018	0.332	−0.003
13	0.346	0.019	0.400	0.100	0.357	0.035	0.311	−0.034	0.299	−0.051	0.328	−0.008
14	0.379	0.069	0.301	−0.049	0.343	0.015	0.318	−0.024	0.308	−0.039	0.347	0.020
15	0.381	0.071	0.355	0.033	0.309	−0.036	0.328	−0.009	0.377	0.065	0.339	0.009
16	0.273	−0.091	0.359	0.039	0.373	0.059	0.349	0.024	0.319	−0.021	0.350	0.025
17	0.388	0.081	0.368	0.051	0.340	0.010	0.305	−0.043	0.339	0.009	0.286	−0.071
18	0.397	0.095	0.373	0.060	0.364	0.046	0.314	−0.029	0.320	−0.020	0.338	0.006
19	0.338	0.006	0.305	−0.043	0.335	0.003	0.389	0.084	0.309	−0.036	0.295	−0.058
20	0.360	0.040	0.395	0.093	0.333	−0.001	0.325	−0.013	0.308	−0.039	0.287	−0.070
**Summary**	**0.368** **±0.049**	**0.050 (0.007)**	**0.366** **±0.047**	**0.045 (0.007)**	**0.350** **±0.020**	**0.025 (0.007)**	**0.344** **±0.033**	**0.014 (0.007)**	**0.329** **±0.023**	**-0.008 (0.007)**	**0.325** **±0.027**	**-0.016 (0.007)**

Accuracy is reported as a proportion and individual values are rounded to three decimal places. In the final row, accuracy is summarized as the across-subject mean ± standard deviation (SD), whereas Cohen's κ is reported as the corresponding pooled estimate with its standard error in parentheses, κ_pooled_ (SE).

This ablation evaluates the Alpha, Beta, and Gamma bands separately for 16- and 64-channel FPGA-synchronized EEG, without centroid construction or PCA.

Mean accuracies range from 0.325 to 0.368, with pooled κ values from −0.016 to 0.050, indicating that no individual band provides robust decoding under this configuration.

#### 3.3.5 Group 4: partial modular combinations (PCA-only and centroid-only)

To test whether partial combinations of our algorithmic modules could bridge the performance gap, Group 4 evaluated sub-band data paired strictly with either PCA alone or the Centroid-Based Correlation algorithm alone (without combining both in sequence).

As detailed in Table 4, sub-bands paired with PCA-only yielded mean accuracies of 35.10% (16 channels) and 34.40% (64 channels), while sub-bands paired with the centroid-only configuration yielded 34.00% (16 channels) and a degenerate chance-level performance of 33.30% (κ_pooled_ = 0.000) in the 64-channel uncompressed space due to distance concentration and feature redundancy. These findings explicitly demonstrate that partial module combinations fail independently.

**Table 4:** Ablation group 4: decoding performance with PCA-only vs. centroid-only feature processing on FPGA-synchronized subband EEG.

Subject	FPGA + Subband + PCA				FPGA + Subband + Centroid			
	16CH		64CH		16CH		64CH	
	Accuracy	Kappa (κ)	Accuracy	Kappa (κ)	Accuracy	Kappa (κ)	Accuracy	Kappa (κ)
1	0.337	0.005	0.317	−0.025	0.320	−0.020	0.333	0.000
2	0.308	−0.039	0.324	−0.014	0.291	−0.064	0.333	0.000
3	0.398	0.098	0.408	0.113	0.330	−0.005	0.333	0.000
4	0.360	0.040	0.370	0.055	0.351	0.026	0.333	0.000
5	0.375	0.063	0.406	0.109	0.319	−0.021	0.333	0.000
6	0.319	−0.021	0.352	0.028	0.327	−0.010	0.333	0.000
7	0.381	0.071	0.393	0.089	0.335	0.003	0.333	0.000
8	0.366	0.049	0.334	0.001	0.383	0.075	0.333	0.000
9	0.343	0.014	0.349	0.024	0.380	0.070	0.333	0.000
10	0.442	0.163	0.396	0.094	0.369	0.054	0.333	0.000
11	0.348	0.023	0.329	−0.006	0.404	0.106	0.333	0.000
12	0.371	0.056	0.334	0.001	0.354	0.031	0.333	0.000
13	0.348	0.021	0.328	−0.009	0.318	−0.023	0.333	0.000
14	0.303	−0.046	0.288	−0.068	0.358	0.038	0.333	0.000
15	0.329	−0.006	0.337	0.005	0.337	0.005	0.333	0.000
16	0.327	−0.010	0.304	−0.044	0.291	−0.064	0.333	0.000
17	0.298	−0.054	0.343	0.015	0.308	−0.038	0.333	0.000
18	0.348	0.023	0.328	−0.009	0.339	0.009	0.333	0.000
19	0.336	0.004	0.306	−0.041	0.318	−0.023	0.333	0.000
20	0.384	0.076	0.331	−0.004	0.372	0.058	0.333	0.000
**Summary**	**0.351** **±0.035**	**0.025 (0.007)**	**0.344** **±0.034**	**0.013 (0.007)**	**0.340** **±0.031**	**0.011 (0.007)**	**0.333** **±0.000**	**0.000 (0.007)**

Accuracy is reported as a proportion and individual values are rounded to three decimal places. In the final row, accuracy is summarized as the across-subject mean ± standard deviation (SD), whereas Cohen's κ is reported as the corresponding pooled estimate with its standard error in parentheses, κ_pooled_ (SE). This ablation compares PCA-only and centroid-only feature processing after subband decomposition for 16- and 64-channel FPGA-synchronized EEG. For 64CH, centroid without PCA, all subjects yielded an accuracy of 0.333 and κ = 0, indicating degenerate classification at the theoretical chance level rather than a predicted class probability. This pattern is consistent with classifier collapse in an uncompressed, high-dimensional centroid space; dimensionality-related effects such as redundancy, distance concentration, or poor conditioning may contribute, although this table alone does not establish the sole cause.

#### 3.3.6 Group 5: full-framework and spatial optimization synergy, and ablation study summary

Finally, Group 5, As detailed in Table 5, represents our complete proposed framework, combining FPGA sub-millisecond synchronization, sub-band decomposition, the Centroid-Based Correlation Algorithm, and PCA dimensionality reduction.

**Table 5:** Ablation group 5: full-framework decoding performance using 16 selected vs. all 64 electrodes.

Subject	FPGA + Subband + Centroid + PCA			
	16CH SELECTED		64CH ALL	
	Accuracy	Kappa (κ)	Accuracy	Kappa (κ)
1	0.700	0.550	0.422	0.133
2	0.667	0.500	0.388	0.081
3	0.733	0.600	0.444	0.167
4	0.700	0.550	0.400	0.100
5	0.667	0.500	0.427	0.140
6	0.700	0.550	0.427	0.140
7	0.667	0.500	0.400	0.100
8	0.667	0.500	0.411	0.116
9	0.667	0.500	0.414	0.121
10	0.667	0.500	0.417	0.125
11	0.667	0.500	0.414	0.121
12	0.700	0.550	0.419	0.128
13	0.717	0.575	0.423	0.135
14	0.667	0.500	0.400	0.100
15	0.667	0.500	0.407	0.111
16	0.667	0.500	0.397	0.095
17	0.667	0.500	0.394	0.092
18	0.633	0.450	0.415	0.122
19	0.667	0.500	0.406	0.109
20	0.667	0.500	0.414	0.121
**Summary**	**0.677** **±0.023**	**0.514 (0.013)**	**0.412** **±0.013**	**0.118 (0.019)**

Accuracy is reported as a proportion and individual values are rounded to three decimal places. In the final row, accuracy is summarized as the across-subject mean ± standard deviation (SD), whereas Cohen's κ is reported as the corresponding pooled estimate with its standard error in parentheses, κ_pooled_ (SE).

This table evaluates the complete FPGA + subband + centroid + PCA framework using either 16 neurophysiologically selected channels or all 64 channels.

The higher accuracy and pooled κ obtained with the selected subset indicate better decoding under this configuration; however, this comparison alone does not determine whether the gain arises primarily from reduced dimensionality, lower noise, or greater spatial relevance.

When evaluated across all 64 electrodes, the full framework achieves an accuracy of 41.20% (κ_pooled_ = 0.118). However, when integrated with our neurophysiologically driven 16-channel spatial optimization subset, the synergistic effect of these modules elevates the global mean accuracy to a more robust 67.70% (κ_pooled_ = 0.514 and Δ+34.5pp) (Table 6, Group 5). This systematic ablation explicitly demonstrates that optimal single-trial decoding relies entirely on the non-linear synergy between microsecond-bounded hardware synchronization, multi-scale spectral decomposition, centroid template correlation, and targeted spatial filtering, as demonstrated by the impact of the Full Framework on the 16 electrode configuration whose performance clearly stands out in the heat map (bright yellow on Table 6).

**Table 6:** Ablation matrix: component-wise performance contribution—heatmap representation.

Component	Channels	Accuracy		κ		Gain
		Mean	SD	Pooled	SE	(pp)
Baseline	16	0.332	0.034	−0.001	0.007	–
+ FPGA Sync	16	0.359	0.033	0.035	0.007	↑2.7
+ Alpha band	16	0.368	0.049	0.050	0.007	↑3.6
+ Beta band	16	0.350	0.020	0.025	0.007	↑1.8
+ Gamma band	16	0.329	0.023	−0.008	0.007	↓0.3
+ Subbands + PCA	16	0.351	0.035	0.025	0.007	↑1.9
+ Subbands + Centroid	16	0.340	0.031	0.011	0.007	↑0.8
**Full Framework**	16	**0.677**	**0.023**	**0.514**	**0.013**	**↑34.5**

Rows represent alternative ablation configurations rather than a strictly cumulative sequence.

Gain is the absolute accuracy difference from the baseline. The numerical percentage-point values provide the quantitative comparison.

FPGA synchronization is applied to all configurations except the baseline.

In bold, results for the framework proposed in this paper.

**Gain**: Higher ▭ ▭ ▭ ▭ ▭ ▭ ▭ Lower

As for the number of electrodes, when all 64 electrodes are used with the full framework, accuracy decreases to 41.2% (Δ+8.0pp), demonstrating electrode selection as an adequate tool for avoiding unnecessary noise and to provide targeted detection.

Concerning frequency-band contribution (Group 3) it can be observed that the Alpha band (Alpha band only) provides the best individual performance among the bands analyzed, yielding improvements of up to +3.6pp (36.8% accuracy). In contrast, the Gamma band performs slightly below the software-jitter baseline (−0.3 to −0.7pp).

It is also very noticeable the effect of FPGA Synchronization and Raw Data (Groups 1 and 2) as moving from the software-jitter baseline (Group 1: 33.2%) to raw EEG synchronized by the FPGA (Group 2: approximately 35.7%–35.9%) provides a consistent initial improvement of approximately 2.5 to 2.7 pp, highlighting the importance of temporal stability during acquisition.

On the other hand, the results also point at the effects of Processing Isolation (Group 4), that is, using subbands with PCA or the centroid method in isolation (Group 4) yields moderate and consistent improvements (+0.1 to +1.9pp), confirming that individual components alone do not explain the exponential increase achieved by integrating the full framework in Group 5.

### 3.4 Classification performance

The discriminative capacity of the spatio-temporal features obtained using our framework was assessed using a One-vs-One classification scheme for the three Spanish corner vowels (/a/, /i/, /u/). For this three-class problem, the One-vs-One strategy trains three independent binary classifiers (i.e., /a/ vs. /i/, /a/ vs. /u/, and /i/ vs. /u/) and determines the final class assignment through a majority voting mechanism, ensuring robust pairwise decision boundaries while preventing class-imbalance issues. To rigorously evaluate this architecture, a 5-fold cross-validation with 10 repetitions was employed, with each iteration allocating 80% of the data for training and 20% for testing. The theoretical chance level for this experimental paradigm is 33.3%.

As shown in Table 7, our proposed method—powered by the synergy of FPGA sub-millisecond synchronization, sub-band decomposition, and the Centroid-Based Correlation Algorithm coupled with a Support Vector Machine (SVM) equipped with a Radial Basis Function (RBF) kernel—achieved a grand mean accuracy of 67.70% with κ_pooled_ = 0.514 (SE = 0.013) across all subjects. It is important to clarify our evaluation terminology: while the classifier strictly operates on un-averaged, individual single-trial features, the reported accuracy metrics represent the percentage of correctly classified trials over the total number of test trials for a given subject.

**Table 7:** Descriptive comparison of single-trial vowel-classification performance and qualitative computational cost.

Study	Language	Feature extraction and classifier	Computational cost	Min.	Mean	Max.
Kim et al. (2014)	Korean	MEMD + LDA	High	65.09%	69.67%	74.00%
Fong et al. (2014)	Cantonese	MDS + FLD	High	66.00%	76.00%	86.00%
Choi et al. (2015)	English	CSP + SVM	Mid	50.50%	57.95%	66.90%
Moinnereau et al. (2018)	English	Echo State Network	High	67.70%	83.20%	90.40%
Hons et al. (2026)	German	MI + MLP	Mid	41.38%	76.79%	98.44%
Wang et al. (2026)	Mandarin	EEG-Conformer (Song et al., 2023)	High	—	50.00%	—
Li and Tao (2026)	English	Random Forest	Mid	11.42%	21.47%	28.75%
**Baseline (hardware only)**	**Spanish**	**Standard Classifier**	**Low**	**30.50%**	**35.90%**	**46.50%**
**Proposed method**	**Spanish**	**Centroids + PCA + SVM**	**Low**	**49.20%**	**67.70%**	**83.00%**

Accuracy values are reported as presented in the cited studies and reflect study-specific datasets, class definitions, sample sizes, validation schemes, and aggregation procedures; consequently, the comparison is descriptive rather than a controlled benchmark.

For the proposed method, Min. and Max. are the across-participant means of each participant's minimum and maximum fold accuracy, respectively; the absolute best fold reached 90.00%.

The corresponding definitions may differ in the cited studies. Computational cost is a qualitative relative category based on the online-inference operations described in the text; it does not represent directly measured runtime, FLOPs, memory use, or energy consumption. An em dash indicates that the corresponding value was not reported.

In bold, results for the framework proposed in this paper, hardware only and full framework.

Individual performance exhibited natural inter-subject variance. According to the subject-level analysis in Section 3.5, mean accuracies for the participant-specific models ranged from 63.33% to 73.33%. Across participants, the averages of the minimum and maximum fold accuracies were 49.20% and 83.00%, respectively, while the absolute peak observed in an individual validation fold was 90.00%. These quantities describe different levels of aggregation and should not be interpreted interchangeably.

The computational cost categorized in Table 7 is operationally evaluated based on the algorithmic complexity and operational latency required during the single-trial online inference phase. Methods classified as High Cost involve recursive iterative sifting processes (e.g., MEMD), non-linear state-space updates (e.g., ESN), or deep hybrid architectures that utilize multi-head self-attention mechanisms, such as the EEG-Conformer (Wang et al., 2026; Song et al., 2023), which require substantial GPU resources for real-time processing. Mid Cost frameworks typically necessitate matrix-wide spatial decompositions (e.g., CSP) or ensemble-based and shallow neural architectures like Random Forest and Multilayer Perceptrons (MLP) (Li and Tao, 2026; Hons et al., 2026), which require fewer resources than deep learning but still involve significant feature optimization stages. Conversely, our proposed Centroids+PCA+SVM pipeline operates at a Low Cost, reducing online processing to non-iterative Pearson correlations and linear projections.

The strategic choice of an RBF kernel empowered the classifier to map non-linearly separable neural oscillations into a higher-dimensional feature space. This approach is particularly robust compared to the findings of Li and Tao (2026), whose recent leakage-audited benchmark reported near-chance accuracies (21.47%) for 5-vowel perception, highlighting the extreme difficulty of cross-subject generalization in controlled auditory tasks. Unlike contemporary deep learning models, such as those validated by Wang et al. (2026) on Mandarin tonal-vowels or Hons et al. (2026) on German phonemes, our Centroid-PCA-SVM-RBF pipeline provides a highly efficient and interpretable solution. While Hons et al. (2026) achieved 76.79% accuracy using MI-based feature selection, our method achieves comparable performance with significantly reduced spatial resolution (16 out of 64 electrodes) and lower algorithmic latency, validated on a Spanish linguistic substrate.

### 3.5 Statistical significance, chance-corrected agreement, and generalization

#### 3.5.1 Within-subject significance and binomial validation

The cohort comprised 20 participants, with 300 trials per participant (100 trials per class), except for Subject 3, who completed 150 trials before voluntarily withdrawing during the inter-session rest period. Statistical significance was evaluated for the subject-specific, within-subject classifiers. Under the null hypothesis of random guessing in the balanced three-class problem, the number of correct decisions follows *X*~Binomial(*n, p*_0_) with *p*_0_ = 1/3. For a one-sided exact binomial test at α = 0.05, the critical accuracy was defined aby Equation 17.


$$\begin{array}{l} A_{\text{crit}}\left(n\right)=\frac{1}{n}\min\left\{k:\mathrm{Pr}\left(X\geq k|n,p_{0}\right)\leq \alpha \right\}. \end{array}$$ (17)

This yields *k*_crit_ = 115 correct decisions for *n* = 300, corresponding to *A*_crit_ = 0.3833, and *k*_crit_ = 61 for Subject 3 (*n* = 150), corresponding to *A*_crit_ = 0.4067. The inferential comparison therefore concerns each participant's aggregate out-of-fold performance. The minimum and maximum fold accuracies reported below characterize cross-validation variability and are not compared with the *n* = 300 threshold. A single five-fold test partition contains approximately 60 trials (30 for Subject 3), and repeated partitions are not independent additional observations.

As summarized in Table 8, subject-level mean accuracies ranged from 63.33% to 73.33%, and all exceeded their corresponding exact binomial boundaries (*p* < 0.0001). These results support above-chance decoding for every participant under subject-specific training and testing. By contrast, the hardware-synchronized broadband baseline had a cohort mean of only 35.90% (As reported in Tables 2, 7), and most participant-level baseline accuracies did not exceed the *n* = 300 exact-binomial benchmark.

**Table 8:** Within-subject classification performance and chance-corrected agreement summarized across participants.

Metric	S1	S2	S3	S4	S5	S6	S7	S8	S9	S10	S11	S12	S13	S14	S15	S16	S17	S18	S19	S20	Acc: Mean ±SD κ_pooled_(SE)
**Trials**	300	300	150	300	300	300	300	300	300	300	300	300	300	300	300	300	300	300	300	300	–
**Acc. Min**.	0.47	0.43	0.47	0.57	0.50	0.43	0.50	0.57	0.40	0.50	0.47	0.47	0.50	0.47	0.47	0.50	0.50	0.53	0.53	0.57	0.492 ± 0.046
**κ** **Min**.	0.20	0.15	0.20	0.35	0.25	0.15	0.25	0.35	0.10	0.25	0.20	0.20	0.25	0.20	0.20	0.25	0.25	0.30	0.30	0.35	0.239 (0.014)
**Acc. Mean**	0.70	0.67	0.73	0.70	0.67	0.70	0.67	0.67	0.67	0.67	0.67	0.70	0.72	0.67	0.67	0.67	0.67	0.63	0.67	0.67	0.678 ± 0.023
**κ** **Mean**	0.55	0.50	0.60	0.55	0.50	0.55	0.50	0.50	0.50	0.50	0.50	0.55	0.58	0.50	0.50	0.50	0.50	0.45	0.50	0.50	0.514 (0.013)
**Acc. Max**	0.90	0.83	0.87	0.83	0.80	0.83	0.90	0.83	0.80	0.80	0.87	0.80	0.83	0.83	0.80	0.80	0.77	0.80	0.83	0.87	0.830 ± 0.036
**κ** **Max**.	0.85	0.75	0.80	0.75	0.70	0.75	0.85	0.75	0.70	0.70	0.80	0.70	0.75	0.75	0.70	0.70	0.65	0.70	0.75	0.80	0.744 (0.010)

This table summarizes subject-specific within-subject models; it does not evaluate transfer to unseen participants. Metrics are proportions (e.g., 0.90 = 90%).

Accuracy summaries are the across-participant mean ± standard deviation (SD), whereas Cohen's κ summaries are the pooled estimate κ_pooled_ with its standard error (SE). Min. and Max. denote the lowest and highest observed cross-validation-fold values for each participant and are descriptive measures of fold variability.

The theoretical chance level is 0.333.

*Statistical significance:* The one-sided exact-binomial boundary is 0.383 for *n* = 300 and 0.407 for Subject 3 (*n* = 150).

All subject-level mean accuracies exceed the applicable boundary (*p* < 0.0001).

#### 3.5.2 Across-participant summary of within-subject decoding: chance-corrected agreement

Table 8 aggregates the results of participant-specific classifiers and must therefore be distinguished from cross-subject generalization. At the mean operating point, the pooled chance-corrected agreement was κ_pooled_ = 0.514 (SE = 0.013), indicating consistently positive agreement beyond that expected from the empirical class marginals. The maximum observed fold-level value was κ = 0.850; like the corresponding maximum accuracy, this value describes the best observed validation fold rather than the expected performance for a new participant.

The pooled agreement associated with the per-participant minimum folds remained positive at κ_pooled_ = 0.239 (SE = 0.014), although these minima are descriptive and are not independent inferential replicates. Relative to the software-jitter baseline (κ_pooled_ = −0.001), the complete framework produced a marked increase in chance-corrected agreement. Together with leakage-free cross-validation, this result supports the presence of discriminative within-subject neural structure without implying zero-shot transfer across participants.

#### 3.5.3 Cross-subject generalization and inter-subject phase dynamics

To evaluate zero-calibration generalization to unseen participants, a Leave-One-Subject-Out (LOSO) cross-validation scheme was implemented across all 20 subjects. In contrast to the subject-specific results above, the LOSO analysis yielded a global mean accuracy of 33.08%, effectively matching the 33.33% chance level. The pooled chance-corrected agreement was κ_pooled_ = −0.0036 (SE = 0.0142), with an approximate 95% confidence interval of [−0.0314, 0.0242]. Because this interval includes zero, the cohort-level LOSO result provides no evidence of agreement beyond chance. This concise global result is reported in text rather than in an additional table.

The decline is consistent with inter-subject phase cancellation, a well-documented limitation in zero-calibration EEG decoding. Recent literature, Li et al. (2026) confirms that severe domain shifts and neural heterogeneity typically degrade cross-subject transfer to near-chance levels. Although the FPGA architecture preserves trial-to-trial timing within each participant, differences in cortical geometry, volume conduction, electrode-to-source mapping, and oscillatory phase can reduce the coherence of a centroid constructed across participants. Consequently, fine-grained Beta- and Gamma-band dynamics that are informative within an individual may be attenuated in an unaligned multi-subject feature space.

At the participant level, held-out Subjects 4, 16, 17, and 20 repeatedly achieved accuracies between 36.00% and 38.67% and showed slight positive agreement. These localized deviations are best interpreted as exploratory evidence of heterogeneity in cross-subject transferability; they do not overturn the near-zero pooled κ or establish reliable donor–recipient compatibility. Future work should test subject clustering, donor selection, spatial or phase alignment, and neural-manifold alignment within a prospectively defined cross-subject protocol.

## 4 Discussion

The empirical findings demonstrate that the proposed centroid-based framework decodes perceived natural speech from non-invasive EEG on a *Single-Trial* basis when models are calibrated within each participant, achieving a global mean accuracy of 67.70% and κ_pooled_ = 0.514 (SE = 0.013). The absolute peak observed in an individual validation fold was 90.00% (κ = 0.850). In contrast, LOSO evaluation remained at chance, showing that the current framework supports personalized decoding but not zero-calibration transfer to unseen participants. This distinction defines both the principal contribution and the present generalization boundary of the study.

While the global within-subject mean demonstrates the utility of the architecture, participant-level mean accuracies ranged from 63.33% to 73.33%, and fold-level accuracies ranged from 40.00% to 90.00%. This variability warrants explicit contextualization. Anatomically, individual differences in cortical folding, particularly the orientation of the superior temporal gyrus relative to the scalp, can alter the spatial projection and signal-to-noise ratio of auditory responses. Cognitively, fluctuations in sustained attention and vigilance can also affect phase consistency. Rather than masking this biological variability through aggressive smoothing, the open-box framework preserves participant-specific physiological structure. Importantly, every participant's aggregate within-subject mean exceeded the applicable exact-binomial boundary, although this does not imply that every individual fold was independently significant.

Unlike conventional approaches that isolate *Event-Related Potentials* (ERPs) by collapsing multi-trial data into a static ensemble average, our architecture actively leverages trial-to-trial variability. Traditional ensemble averaging operates under the restrictive assumption that task-related neural components are strictly phase-locked, treating non-phase-locked dynamics and latency jitter as stochastic noise to be attenuated. However, in the context of high-order cognitive processes such as speech perception, this low-pass filtering effect masks crucial time-varying and non-stationary neural architectures. By combining high-precision FPGA hardware synchronization with a sub-band centroid correlation scheme, our framework successfully isolates both the exogenous sensory responses and the fine-grained endogenous cognitive modulations of phonetic content on an epoch-by-epoch basis.

A critical point of discussion is the comparison between our mean classification performance (67.70%) and the higher accuracies frequently reported by contemporary deep learning models (e.g., recurrent reservoir networks or deep convolutional topologies achieving >80%). While our numerical mean is lower, this is a deliberate, necessary, and advantageous trade-off for neuroprosthetic design. Opaque, high-dimensional neural networks evaluated on jitter-prone or loosely synchronized data are highly susceptible to the “Clever Hans” effect—unintentionally classifying non-task-related physiological artifacts, spatial electrode leakage, temporal drift, or stochastic autocorrelations rather than actual language processing. Conversely, our framework delivers a physiologically grounded metric of real phonetic discrimination. As validated by the rigorous intra-fold template isolation implemented during cross-validation, the calculation of class centroids was restricted strictly to the training partition, eliminating any risk of data leakage or “double-dipping” statistical biases. Consequently, our within-subject performance reflects generalization to unseen trials from a calibrated participant, rather than generalization to an unseen participant. Furthermore, as summarized in the low computational cost profiles, our framework bypasses the massive matrix operations and power-hungry optimization cycles typical of deep learning architectures, rendering it uniquely viable for deployment in low-power, real-time BCI edge-computing and wearable devices.

The neurophysiological validity of our framework is heavily reinforced by the spatial optimization that compressed the electrode grid to a 16-channel subset without compromising decoding accuracy. These specific channels—concentrated primarily around the frontotemporal (FT7, FT8), temporal (T7, T8), and occipitoparietal (Iz) configurations—map directly onto the primary and secondary auditory cortices, the superior temporal gyrus (STG), and lateral prefrontal regions involved in speech processing. This spatial alignment demonstrates that our high-precision hardware-driven clock synchronization successfully isolated the exact biological generators of acoustic and phonemic processing, permitting a 75% dimensionality reduction that directly mitigates clinical and computational overhead.

From an acoustic-phonetic perspective, the separability of the vowels /a/, /i/, and /u/ within the decomposed sub-bands (α, β, *andγ*) is highly coherent with speech science. In human perception, these corner vowels are defined by distinct acoustic formants: /a/ features a high F1 frequency, /i/ exhibits a prominent F2 frequency, and /u/ is characterized by low F1 and F2 formants. Our findings imply that the human auditory cortex mirrors these acoustic distances through localized phase-locked and non-phase-locked high-frequency oscillations. Notably, the sub-millisecond deterministic alignment provided by our FPGA sync was the sole enabling factor that allowed the linear summation in the time domain to act as a phase-sensitive coherent filter. Under this condition, trials sharing consistent phoneme-specific phase relationships underwent constructive interference within the centroid *C*_*k*_, preserving the transient Gamma-band dynamics. Conversely, stochastic background noise experienced destructive interference.

The ability to operate effectively using 300 trials per subject (and 150 trials for Subject 3 due to early withdrawal) carries implications for clinical transition. Traditional multi-trial BCIs impose cognitive and physical demands on vulnerable populations, such as individuals with Locked-In Syndrome or Amyotrophic Lateral Sclerosis. Every participant's aggregate within-subject mean exceeded the applicable exact-binomial threshold, supporting reliable personalized decoding at the participant level. However, the chance-level LOSO result shows that participant-specific calibration or an explicit alignment stage remains necessary before the framework can support cross-subject clinical deployment. Future work should also evaluate speaker-held-out and token-held-out validation schemes, in which the EEG trials elicited by specific stimulus speakers or individual audio recordings are reserved exclusively for testing. Such validation would establish whether the decoded neural representation is invariant to speaker identity and token-specific acoustic characteristics, rather than being limited to unseen EEG trials drawn from the same pool of stimulus voices.

In summary, the core technological breakthrough of this work demonstrates that single-trial neural variability is not a stochastic confound to be eliminated, but rather a structured manifold rich in discriminative cognitive information when captured with hardware-level temporal determinism. By striking an optimal balance between high physiological interpretability, computational efficiency, and steady decoding performance, this open-box paradigm overcomes key limitations of both classical grand-averaging and black-box deep learning models.

While the successful classification of the canonical corner vowels (/a/, /i/, /u/) establishes a crucial neurophysiological baseline by validating the system's sensitivity to extreme formant boundaries, we acknowledge this as a foundational proof-of-concept rather than a complete linguistic solution. Future work will focus on enhancing the front-end signal-to-noise ratio (SNR) through online adaptive filtering to further protect baseline validation boundaries under noisy clinical environments. Building upon this foundation, subsequent efforts will scale this framework from isolated static phonemes to the continuous, unconstrained decoding of natural, fluid running speech, serving as a transparent and adaptive stepping stone toward next-generation non-invasive neuroprosthetic devices.

## Data Availability

The dataset presented in this study can be found in online repository. The name of the repository and accession numbe can be found below: https://dx.doi.org/10.21227/bee6-a870.
